# Supplementary material for: Deciphering functional redundancy in the human microbiome
Source: Nat Commun. 2020 Dec 4;11:6217. doi: 10.1038/s41467-020-19940-1 (PMC7719190; doi:10.1038/s41467-020-19940-1)
Supplement: Supplementary file 1 — Supplementary Information [file 41467_2020_19940_MOESM1_ESM.pdf]

Deciphering Functional Redundancy in the Human Microbiome: *Supplementary Information*

Liang Tian, Xu-Wen Wang, Ang-Kun Wu, Yu-Hang Fan, Jonathan Friedman, Amber Dahlin, Matthew K. Waldor, George M. Weinstock, Scott T. Weiss, Yang-Yu Liu*

November 3, 2020

Contents

1 Diversity and redundancy measures based on Hill numbers 3

1.1 Taxonomic diversity . . . 3

1.2 Functional diversity . . . 4

1.3 Functional redundancy . . . 6

2 FR calculation using shotgun metagenomic sequencing data 9

2.1 Using reference genomes . . . 9

2.1.1 Reference GCN . . . 9

2.1.2 Body site-specific GCN . . . 9

2.1.3 Taxonomic profiling of SMS data . . . 10

2.2 Without using reference genomes . . . 10

2.2.1 Construction of GCN . . . 10

2.2.2 *De novo* taxonomic profiling based on co-abundance gene groups . . . 11

3 Null models 13

3.1 Randomizing the GCN . . . 13

3.2 Randomizing the microbial composition . . . 13

*Corresponding author: yyl@channing.harvard.edu

4 Genome evolution model **15**
4.1 Model description 15
4.2 Simulation results 15

5 Supplementary figures **17**

1 Diversity and redundancy measures based on Hill numbers

Various taxonomic and functional diversity measurements have been defined based on Hill number to characterize the diversity of ecological systems. In this section, we first briefly introduce those Hill number based taxonomic and functional diversity measures. In particular, we point out some limitations in the existing definitions of Hill number based functional diversity. We propose alternative definitions that overcome those limitations. Finally, we introduce Hill number based functional redundancy based on the existing Hill number based taxonomic diversity measures and our modified Hill number based functional diversity measures. Note that the proposed measures are not just valid for the analysis of microbiome data. In principle, they can also be used to analyze more general ecological data.

1.1 Taxonomic diversity

Consider a sample of N species with the relative abundance profile given by a vector $\mathbf{p} = [p_1, \cdots, p_N]$. Here the term "species" is used in the general context of ecology, which doesn't necessarily represent the lowest major taxonomic rank. Hill introduced the concept of *effective number of species* [1]. The basic idea is that the taxonomic diversity (of order q) of a given sample with relative abundance profile $\mathbf{p} = [p_1, \cdots, p_N]$ is the same as that of an idealized sample of D equally abundant species with relative abundance profile $\tilde{\mathbf{p}} = [1/D, \cdots, 1/D]$. In other words,

$$\sum_{i=1}^{N} p_i^q = \sum_{i=1}^{D} \left(\frac{1}{D}\right)^q = D^{1-q}. \tag{S1}$$

This offers a parametric class of taxonomic diversity measures defined as follows [1]:

$$\mathrm{TD}_q := \left(\sum_{i=1}^{N} p_i^q\right)^{\frac{1}{(1-q)}} \quad \text{for} \quad q \neq 1. \tag{S2}$$

and

$$\mathrm{TD}_1 := \lim_{q \to 1} \mathrm{TD}_q = \exp\left(-\sum_{i=1}^{N} p_i \log p_i\right). \tag{S3}$$

A few special cases:

- $q = 0$, $\mathrm{TD}_0 = N$ is nothing but the *species richness*;
- $q = 1$, TD_1 is the exponential of the *Shannon entropy* (a.k.a. *Shannon index*);
- $q = 2$, $\mathrm{TD}_2 = 1/\sum_{i=1}^{N} p_i^2$ is the *inverse Simpson index* (a.k.a. *Simpson diversity*).

Note that the *Gini-Simpson index* (GSI) used in the main text is related to TD_2 as follows:

$$\mathrm{GSI} := 1 - \sum_{i=1}^{N} p_i^2 = 1 - \frac{1}{\mathrm{TD}_2}. \tag{S4}$$

1.2 Functional diversity

Consider a sample of N species with the relative abundance profile $\mathbf{p} = [p_1, \cdots, p_N]$, and pair-wise functional distance matrix $\Delta = (d_{ij}) \in \mathbb{R}^{N\times N}$ with $d_{ii} = 0$ for all $i = 1, \cdots, N$, and $d_{ij} = d_{ji} \geq 0$ for all $i \neq j$. Recently, Chiu *et al.* extended the notion of Hill numbers to incorporate species pairwise functional distances and introduced the so-called functional Hill number [2]. In this framework, they derived the functional Hill number as follows:

$$\sum_{i=1}^{N} \sum_{j=1,j\neq i}^{N} d_{ij}(p_i p_j)^q = \sum_{i=1}^{D} \sum_{j=1,j\neq i}^{D} Q^*(\frac{1}{D}\frac{1}{D})^q \tag{S5}$$

where

$$Q^* \equiv \frac{\sum_{i=1}^{N} \sum_{j=1,j\neq i}^{N} d_{ij} p_i p_j}{\sum_{i=1}^{N} \sum_{j=1,j\neq i}^{N} p_i p_j} \tag{S6}$$

denotes the average pair-wise distance weighted by their abundances. Note that when different species are equally distinct with a constant pairwise distance, Q^* must be equal to this constant. The functional Hill number D can be interpreted as "the effective number of equally abundant and (functionally) equally distinct species" with a constant distance Q^* for all species pairs (i, j) with $i \neq j$.

We notice that there are two drawbacks in this framework: (i) The functional Hill number D can not be explicitly solved from Eq.S5; (2) When all different species in the assemblage are equally distinct (i.e., $d_{ij} = Q^*$ for all $i \neq j$), the functional Hill number D should reduce to the traditional or taxonomic Hill number. But according to (Eq.S5) and Eq.S6, we have

$$\sum_{i=1}^{N} \sum_{j=1,j\neq i}^{N} (p_i p_j)^q = (D-1)D^{1-2q}. \tag{S7}$$

Apparently, D dose not reduce to TD_q in (Eq.S2) or (Eq.S3).

In order to overcome those two drawbacks, we introduce a new pair-wise functional distance matrix

$$\Delta' = (d'_{ij}) = \begin{pmatrix} \lambda_1 & d_{12} & d_{13} & \cdots & d_{1N} \\ d_{21} & \lambda_2 & d_{23} & \cdots & d_{2N} \\ d_{31} & d_{32} & \lambda_3 & \cdots & d_{3N} \\ \vdots & \vdots & \vdots & \ddots & \vdots \\ d_{N1} & d_{N2} & d_{N3} & \cdots & \lambda_N \end{pmatrix}, \tag{S8}$$

where d_{ij} denotes the original functional distance between species-i and j, with $d_{ij} = d_{ji} \geq 0$ ($i \neq j$), and

$$\lambda_i := \frac{\sum_{j\neq i}^{N} d_{ij}}{N-1}, \tag{S9}$$

is the average functional distance between species-i and all the other species. Note that, when different species are equally distinct with a constant pairwise distance, λ is equal to this constant.

Equipped with the modified distance matrix Δ', we can now derive the functional Hill number as follows:

$$\sum_{i=1}^{N}\sum_{j=1}^{N} d'_{ij}(p_i p_j)^q = \sum_{i=1}^{D}\sum_{j=1}^{D} Q'(\frac{1}{D}\frac{1}{D})^q \tag{S10}$$

where

$$Q' := \sum_{i=1}^{N}\sum_{j=1}^{N} d'_{ij} p_i p_j. \tag{S11}$$

Note that Q' shares almost the exact form as *Rao's quadratic entropy* $Q := \sum_{i=1}^{N}\sum_{j=1}^{N} d_{ij} p_i p_j$, a classic functional diversity measure that characterizes the mean distance between any two randomly chosen taxa in the sample [3]. From (Eq.S10), we can derive a parametric class of functional Hill numbers:

$$D_q(Q') := \left(\sum_{i=1}^{N}\sum_{j=1}^{N} \frac{d'_{ij}}{Q'}(p_i p_j)^q\right)^{\frac{1}{2(1-q)}} \quad \text{for} \quad q \neq 1, \tag{S12}$$

and

$$D_1(Q') := \lim_{q\to 1} D_q(Q') = \exp\left[-\frac{1}{2}\sum_{i=1}^{N}\sum_{j=1}^{N} \frac{d'_{ij}}{Q'} p_i p_j \log(p_i p_j)\right]. \tag{S13}$$

The functional Hill number of order q, i.e., $D_q(Q')$, can be interpreted as "the effective number of equally abundant and (functionally) equally distinct species" with a constant distance Q' for all species pairs. Thus, if $D_q(Q') = v$, it just means that the functional diversity (of order q) of a given real sample with relative abundance profile $\mathbf{p} = [p_1, \cdots, p_N]$ and pair-wise functional distance matrix Δ' is the same as that of an idealized sample containing v equally abundant species (i.e., $\tilde{\mathbf{p}} = [1/v, \cdots, 1/v]$), and a $v \times v$ distance matrix with constant pair-wise functional distance Q', i.e.,

$$\begin{pmatrix} Q' & Q' & Q' & \cdots & Q' \\ Q' & Q' & Q' & \cdots & Q' \\ Q' & Q' & Q' & \cdots & Q' \\ \vdots & \vdots & \vdots & \ddots & \vdots \\ Q' & Q' & Q' & \cdots & Q' \end{pmatrix}_{v\times v}. \tag{S14}$$

Hence, the functional diversity of a real sample can be defined as the column (or row) sum of the above idealized $v \times v$ constant matrix, yielding

$$\mathrm{FD}_q(Q') := D_q(Q') \cdot Q' = \left(\sum_{i=1}^{N} \sum_{j=1}^{N} \frac{d'_{ij}}{Q'} (p_i p_j)^q \right)^{\frac{1}{2(1-q)}} \cdot Q' \quad \text{for} \quad q \neq 1, \tag{S15}$$

and

$$\mathrm{FD}_1(Q') := D_1(Q') \cdot Q' = \exp\left[-\frac{1}{2} \sum_{i=1}^{N} \sum_{j=1}^{N} \frac{d'_{ij}}{Q'} p_i p_j \log(p_i p_j) \right] \cdot Q'. \tag{S16}$$

A few special cases:

- If all the different species in the sample are equally distinct, i.e., $d'_{ij} = Q'$ for all species pairs (i, j), $i \neq j$; and $d'_{ii} = \lambda_i = Q'$, then the functional Hill number $D_q(Q')$ reduces to the ordinary Hill number TD_q, and $\mathrm{FD}_q(Q') = \mathrm{TD}_q \cdot Q'$.

- If all the species in the sample are equally aundant, i.e., $p_i = 1/N$ for $i = 1, \cdots, N$, then for any distance matrix Δ', we have $Q' = N^{-2} \sum_{i=1}^{N} \sum_{j=1}^{N} d'_{ij}$, and $D_q(Q') = N$ for all orders of q, and $\mathrm{FD}_q(Q') = D_q(Q') \cdot Q' = N^{-1} \sum_{i=1}^{N} \sum_{j=1}^{N} d'_{ij}$.

1.3 Functional redundancy

In the literature of ecology, the *functional redundancy* (FR) of a sample is often considered to be the part of the taxonomic diversity (TD) that can not be explained by the functional diversity (FD) [4, 5, 6]. Hence FR is typically defined to be the difference between TD and FD:

$$\mathrm{FR} := \mathrm{TD} - \mathrm{FD}. \tag{S17}$$

In Ref. [4, 5], TD was chosen to be the Gini-Simpson index: $\mathrm{GSI} := 1 - \sum_{i=1}^{N} p_i^2$, and FD be Rao's quadratic entropy $Q := \sum_{i=1}^{N} \sum_{j=1}^{N} d_{ij} p_i p_j$. Hence,

$$\mathrm{FR} = \mathrm{GSI} - Q = 1 - \sum_{i=1}^{N} p_i^2 - \sum_{i=1}^{N} \sum_{j=1}^{N} d_{ij} p_i p_j = \sum_{i=1}^{N} \sum_{j=1}^{N} (1 - d_{ij}) p_i p_j. \tag{S18}$$

Consider $d_{ij} \in [0, 1]$. We can verify that FR defined in Eq.(S18) takes the range $[0, 1]$.

- When the species are completely different in their functions, i.e., for all pairs $i \neq j$, $d_{ij} = 1$; and $d_{ii} = 0$, then $Q = \sum_{i=1}^{N} \sum_{j=1, j\neq i}^{N} p_i p_j = \sum_{i=1}^{N} p_i(1 - p_i) = \mathrm{GSI}$, hence $\mathrm{FR} = 0$. This makes perfect sense, because all the different species are functionally different, hence the functional redundancy of this sample (community) is zero.

- When all the species are functionally identical: $d_{ij} = 0$ for $i, j = 1, \cdots, N$, then $Q = 0$, and FR = GSI. In this case, the functional redundancy of this sample (community) is maximized and equal to the Gini-Simpson index of taxa diversity.

- When all the species are functionally identical: $d_{ij} = 0$ for $i, j = 1, \cdots, N$, and in addition the number of species N is very large and the species are equally abundant $p_i = 1/N \to 0$, then FR = GSI $\to 1$.

Now we consider the Hill number based taxonomic diversity TD_q and functional diversity $\mathrm{FD}_q(Q')$, and define a parametric class of functional redundancy:

$$\mathrm{FR}_q(Q') := \mathrm{TD}_q - \mathrm{FD}_q(Q'). \tag{S19}$$

For $q \neq 1$,

$$\mathrm{FR}_q(Q') := \left[\sum_{i=1}^{N} p_i^q\right]^{\frac{1}{(1-q)}} - \left[\sum_{i=1}^{N}\sum_{j=1}^{N} \frac{d'_{ij}}{Q'}(p_i p_j)^q\right]^{\frac{1}{2(1-q)}} \cdot Q'. \tag{S20}$$

For $q = 1$,

$$\mathrm{FR}_1(Q') := \exp\left[-\sum_{i=1}^{N} p_i \log p_i\right] - \exp\left[-\frac{1}{2}\sum_{i=1}^{N}\sum_{j=1}^{N} \frac{d'_{ij}}{Q'} p_i p_j \log(p_i p_j)\right] \cdot Q'. \tag{S21}$$

Consider two extreme cases:

(1) Zero redundancy: $d_{ij} = 1$ for all species pair $i \neq j$. Then, we have $d'_{ij} = 1$ for all i and j, and $Q' = 1$. For $q \neq 1$

$$\mathrm{FD}_q(Q) = \left[\sum_{i=1}^{N}\sum_{j=1}^{N} (p_i p_j)^q\right]^{\frac{1}{2(1-q)}} = \left[\left(\sum_{i=1}^{N} p_i^q\right) \cdot \left(\sum_{j=1}^{N} p_j^q\right)\right]^{\frac{1}{2(1-q)}} = \mathrm{TD}_q.$$

For $q = 1$,

$$\begin{aligned}
\mathrm{FD}_1(Q) &= \exp\left[-\frac{1}{2}\sum_{i=1}^{N}\sum_{j=1}^{N} p_i p_j \log(p_i p_j)\right] \\
&= \exp\left[-\frac{1}{2}\sum_{i=1}^{N}\sum_{j=1}^{N} p_i p_j \log(p_i) - \frac{1}{2}\sum_{i=1}^{N}\sum_{j=1}^{N} p_i p_j \log(p_j)\right] \\
&= \exp\left[-\frac{1}{2}\sum_{i=1}^{N} p_i \log(p_i) - \frac{1}{2}\sum_{j=1}^{N} p_j \log(p_j)\right] \\
&= \mathrm{TD}_1.
\end{aligned} \tag{S22}$$

Therefore, for $d'_{ij} = 1$ we have $\mathrm{FR}_q(Q') = \mathrm{TD}_q - \mathrm{FD}_q(Q') = 0$.

(2) Complete redundancy: $d_{ij} = 0$ for all species pair $i \neq j$, then $d'_{ij} = 0$, $Q' = 0$, $\mathrm{FD}_q(Q') = D_q(Q') \cdot Q' = 0$, and $\mathrm{FR}_q(Q') = \mathrm{TD}_q$ is maximized.

2 FR calculation using shotgun metagenomic sequencing data

From shotgun metagenomic sequencing (SMS) data of microbiome samples, we have two fundamentally different methods to calculate the FR. One relies on reference genomes. The other doesn't.

2.1 Using reference genomes

2.1.1 Reference GCN

We construct the reference GCN based on the HMP reference genomes, which include most of the representative microbes from human body sites. In our study, we consider the bacterial and archaeal genomes from the HMP reference genomes downloaded from the Integrated Microbial Genomes and Microbiome (IMG/M) database, more specifically, the IMG/M-HMP data mart [7]. In total, there are 1,555 strains (genomes) annotated by 7,210 KEGG orthologs (KOs) or 4,371 Clusters of Orthologous Groups (COGs). In order to reduce the culturing and sequencing bias for certain species (e.g., *Escherichia coli* that have many strains), we randomly chose a representative strain (genome) for each species. This results in a reference GCN of 796 species and 7,105 KOs (or 4,371 COGs). In main text Fig. 2a, this reference GCN is visualized at the order level for taxa nodes and the KEGG super-pathway level for functional nodes. The genome of each order is obtained by averaging the genomes of all the species belonging to that order. The bar height of each order corresponds to average genome size of the species belonging to that order. The thickness of a link connecting an order and a KEGG super-pathway is proportional to the number of KOs that belong to that super-pathway, as well as the genomes of species in that order. The network properties of this reference GCN are shown in main text Fig. 2b-e, where each taxon node represents a species, and each functional node represents a KO.

2.1.2 Body site-specific GCN

For each metagenomic sample, its taxonomic profile at the strain level is inferred by using MetaPhlAn2 [8]. For the strains identified in each sample, we first look up the HMP reference genomes in the IMG/M-HMP data mart. If all the strains are included in this data mart, we can directly construct the GCN for this sample. If some of the strains in the sample are not included in the IMG/M-HMP data mart, we will refer to the IMG/M data mart in the IMG data base. The IMG/M data mart includes more than 80,000 genomes for bacteria, archaea, eukarya, viruses, and so on. For our study, the annotated genomes of all the strains can be found and downloaded from the IMG/M data mart. In this way, the GCN for each sample can be constructed. By consider the pool of strains that appear in all the samples from a

certain body site, we can naturally construct a body site-specific GCN. This is equivalent to merging all the sample-specific GCNs for samples from the same body site to a large network. Consequently, in our calculations (Fig. 3a-b in the main text), for each body site, we just randomized the body site-specific GCN, rather than those sample-specific GCNs (which are just the subgraphs of the body site-specific GCN).

2.1.3 Taxonomic profiling of SMS data

There are two different approaches for taxonomic profiling of SMS data. The first one relies on comparisons to reference genomes, and the second one is *de novo* identification and assembly of genomes without using reference genomes. In this work, to check if our main results are sensitive to the taxonomic profiling approaches, we used a representative pipeline of each approach to perform taxonomic profiling of SMS data. Here we talk about the first approach. The second approach will be described in Sec.2.2.

MetaPhlAn is a computational tool for profiling the composition of microbial communities from SMS data. It uses clade-specific marker genes to unambiguously assign reads to microbial clades. The first version of MetaPhlAn (MetaPhlAn1 [9]) relies on unique clade-specific marker genes identified from ∼3,000 reference genomes, allowing species-level resolution for bacterial and archaeal organisms. The enhanced or second version of MetaPhlAn (MetaPhlAn2 [8]) relies on ∼1M unique clade-specific marker genes identified from ∼17,000 reference genomes (∼13,500 bacterial and archaeal, ∼3,500 viral, and ∼110 eukaryotic). It complements the original species-level profiling method with a system for profiling the composition of microbial communities (Bacteria, Archaea, Eukaryotes and Viruses) from SMS data with strain-level resolution. In our study, we used MetaPhlAn2 with its default settings. Especially, the sensitivity argument for the mapping process is set to "very-sensitive". More information can be found at http://huttenhower.sph.harvard.edu/metaphlan2. Note that the samples with less than 5 strains with known genomes were excluded from downstream analysis.

2.2 Without using reference genomes

2.2.1 Construction of GCN

Recently, Nielsen *et al.* developed a powerful method to interpret metagenomic data at the level of individual genomes without relying on reference genomes [10]. In this method, individual genomes are assembled based on clustering of co-abundant genes. First, multiple metagenomic samples (e.g., the gut microbiome samples of hundreds of people) are sequenced, and reads are assembled into genes. For each sample, the abundance of each gene is quantified by the coverage of metagenomic reads. Then, those groups of genes that co-vary in abundance, i.e., show similar abundance across all the metagenomic samples, are

identified. A key idea of this method is that those co-abundant gene groups (CAGs) are inferred as belonging to the same genome of a biological entity such as species. Hence, a natural by-product of this method is the GCN. Both sample-specific and body site-specific GCNs can be naturally constructed from those CAGs.

2.2.2 *De novo* taxonomic profiling based on co-abundance gene groups

Segregating a metagenome into groups of genes that have similar abundance cross a collection of samples allows us to identify microbial genomes without using reference sequences [10].

First, a nonredundant metagenomic gene catalog was constructed. To achieve that, one can employ the MOCAT pipeline [11] to assemble the raw sequencing reads from the MetaHIT metagenomic samples into scaftigs (i.e., continuous sequences within scaffolds). In particular, one can use FastX (Http://http://hannonlab.cshl.edu/fastx_toolkit/index.html) to filter raw sequencing reads with a quality cutoff of 20, and discarded reads shorter than 30 bp. Then SOAPdenovo (version 1.05) [12] can be used to assemble high-quality reads into scaftigs. One can then use MetaGeneMark [13] to predict genes on those scaftigs longer than 500 bp, and then use BLAT [14] to cluster the predicted genes from all samples. Those genes with identity greater than 95% and covering more than 90% of the shorter genes will be clustered together. Finally, one can discard cluster representatives shorter than 100 bp, resulting in a set of nonredundant genes. One can further discard those genes that were likely originated from human, animals or plants from this set of nonredudant genes. The remaining genes form the reference gene catalog.

Second, the abundances of the reference genes can be quantified. One can use the screen function in MOCAT [11] to map high-quality reads to the reference gene catalog. One can subsequently filter those mapped reads using a 30-bp length and 95% identity cutoff. The soap.coverage script [15] is then used to calculate the gene-length normalized base counts. For those samples with 11M or more sequence reads, one can randomly draw 11M sequence reads without replacement. Those randomly drawn reads are mapped to the gene catalog, and the number of reads is counted to form a downsized depth or abundance matrix. One can use the 11M downsized depth matrix to estimate the abundance of co-variance gene groups (CAGs).

Finally, co-abundance clustering is performed. This is achieved by canopy clustering, a very simple and fast method for grouping objects into clusters [16]. Among those not-yet-clustered genes, one can pick a seed gene and segregate those genes whose abundance profiles are within a fixed distance from that of the seed gene into the seed canopy. The distance can be chosen as Person correlation coefficient (PCC) > 0.9 and Spearman's rank correlation coefficient > 0.6. This process is performed iteratively to obtain many canopies. For each canopy, its median abundance profile is calculated. Any two canopies are close enough, i.e., their median abundance profiles are within a distance of 0.97 PCC from each other are

merged. The resulting canopies will be further tested based on the following criteria: (1) it has two or less genes; (2) any three samples constitute 90% or more of the total canopy abundance signal; (3) the median abundance profile is detected in less than four samples; (4) one sample makes up 90% of the total signal. Only those canopies that pass all these criteria are called CAGs. And those CAGs that have more than 700 genes are referred to as metagenomic species (MGS) or just species.

3 Null models

3.1 Randomizing the GCN

To identify the structural features of the GCN that detemine the functional redundancy and functional diversity of microbial communities, we randomized the GCN using four different randomization schemes, yielding four different null-GCN models:

- (Null-GCN-1) Complete randomization. We keep the number of taxa (N) and number of genes (M) unchanged, but otherwise completely rewire the links between taxa and genes. The randomized GCN displays Poisson-like degree distributions for both taxon- and gene-nodes.

- (Null-GCN-2) Taxon-degree preserving randomization. We keep N, M, and the degree of each taxon node unchanged, but selects randomly the gene nodes that link to each taxon. The randomized GCN will have exactly the same taxon degree distribution as the original GCN, but a Poisson-like gene degree distribution.

- (Null-GCN-3) Gene-degree preserving randomization. Here we keep N, M, and the degree of each gene unchanged, but selects randomly the taxa that link to each gene. The randomized GCN will have exactly the same gene degree distribution as the original GCN, and a Poisson-like taxon degree distribution.

- (Null-GCN-4) Gene-degree and taxon-degree preserving randomization. Here we keep N, M, and the degree of each gene and the degree of each taxon unchanged, but randomly rewire the links between taxon nodes and gene nodes. This is achieved as follows. In each step, we randomly pick two links (i, a) and (j, b), where the taxon nodes $i \neq j$ and the gene nodes $a \neq b$. Then we rewire those two links by switching their taxon nodes (or gene nodes), yielding two new links (i, b) and (j, a). In case gene-a (gene-b) already exists in the genome of taxon-j (taxon-i), we simply increase its copy number by one. We repeat this process until every link has been rewired for at least once. The gene degree distribution and the taxon degree distribution of the randomized GCN will be exactly the same as those of the original GCN.

Note that the weight of each link in the original GCN is the gene copy number, which is a non-negative integer. In all the randomization processes, we treat the weighted link as multiple links of unit weight.

3.2 Randomizing the microbial composition

To identify how the microbial composition contributes to the functional redundancy and functional diversity, we randomized taxonomic abundance profile using three different randomization schemes, yielding three different null-composition models:

- (Null-composition-1) Microbial assemblage randomization. For each sample, we randomly choosing the same number of strains from the strain pool but keep the strain abundance profile unchanged to generate a randomized profile. Specifically, for the abundance table of each body site, we randomly permute the table elements along each column (each sample) to generate a randomized abundance table.

- (Null-composition-2) Strain abundance randomization I. For each sample, we keep the microbial assemblage or collection unchanged, and randomize their abundance. Specifically, in this scheme, we randomize the abundance table of each body site through random permutation of non-zero abundance along each column (each sample) across different strains.

- (Null-composition-3) Strain abundance randomization II. For each sample, we keep the microbial assemblage or collection unchanged, and randomize their abundance. Specifically, in this scheme, we randomize the abundance table of each body site through random permutation of non-zero abundance along each row (each strain) across different samples.

Note that for Null-composition-2 and Null-composition-3, the randomized abundance tables are not normalized even though the original abundance table are normalized. Therefore, after the randomizations, we normalize them again before the calculations of FR, FD, and TD.

4 Genome evolution model

4.1 Model description

To reproduce the key topological features of the real GCN (e.g., highly nested structure, fat-tailed gene degree distribution, etc), we developed a simple genome evolution model, which consists of the following steps:

Step-1 At time $t = 0$, we set the initial GCN as a random bipartite graph with n_0 species and m_0 genes. A species is randomly connected to a gene with probability p_0. In our simulation, we chose $n_0 = 500$, $m_0 = 200$, and $p_0 = 0.8$.

Step-2 At each time step $t > 0$, a species i is chosen with probability: $p_i = \frac{k_i^h}{\sum_j k_j^h}$, where $h \geq 0$ is a tuning parameter, and k_i is the degree (i.e., the genome size) of species i. Then the genome of species i will be updated based on one of the following three events:

Event-I: With probability q_{HGT}, **horizontal gene transfer (HGT)** occurs. A donor species is randomly selected. Then one of the donor species' genes a that is not in the genome of the recipient species is transferred to the recipient species' genome. In the GCN, a link is added between this gene node and the recipient species node.

Event-II: With probability q_{gg}, **gene gain** occurs. A new gene is added to the genome of species i. In the GCN, we connect this new gene node to the species node i.

Event-III: With probability q_{gl}, **gene loss** occurs. A gene a in the genome of species i is randomly selected to be lost. In the GCN, a link is deleted between this gene node and the species node i.

Step-3 Repeat Step-2 until the system reaches a desired time step. In our simulation, we typically let the GCN evolve for 5×10^5 steps.

Note that the three events in Step-2 are not fully independent, because $q_{\mathrm{HGT}}+q_{\mathrm{gg}}+q_{\mathrm{gl}} = 1$. Moreover, for any $h > 0$ species with larger genome sizes are more likely to be chosen, implying that selection pressure is implicitly considered in our model. The case $h = 0$ corresponds to the neutral model, where all the species have equal probability to be chosen to update their genomes.

4.2 Simulation results

We have systematically tested that our simple genome evolution model can evolve into a relatively stable state (Supplementary Figure 11), where the constructed GCN displays all the desired topological features as observed in the real GCN, i.e., highly nested structure

(NODF ~ 0.7), fat-tailed gene degree distribution, Poisson-like species degree distribution, and unimodal functional distance distribution. Those key topological features can also be reproduced by other parameter setting, e.g., with different gene gain rate $q_{\rm gg}$ (Supplementary Figure 12). Finally, we point out that if $n = 0$ or n is too large, both the incidence matrix of GCN and the functional distance distribution $P(d_{ij})$ will be quite different from that observed in the real GCN. This implies that moderate selection pressure is needed to reproduce key topological features of the GCN (Supplementary Figure 13), and consequently favor high functional redundancy.

5 Supplementary figures

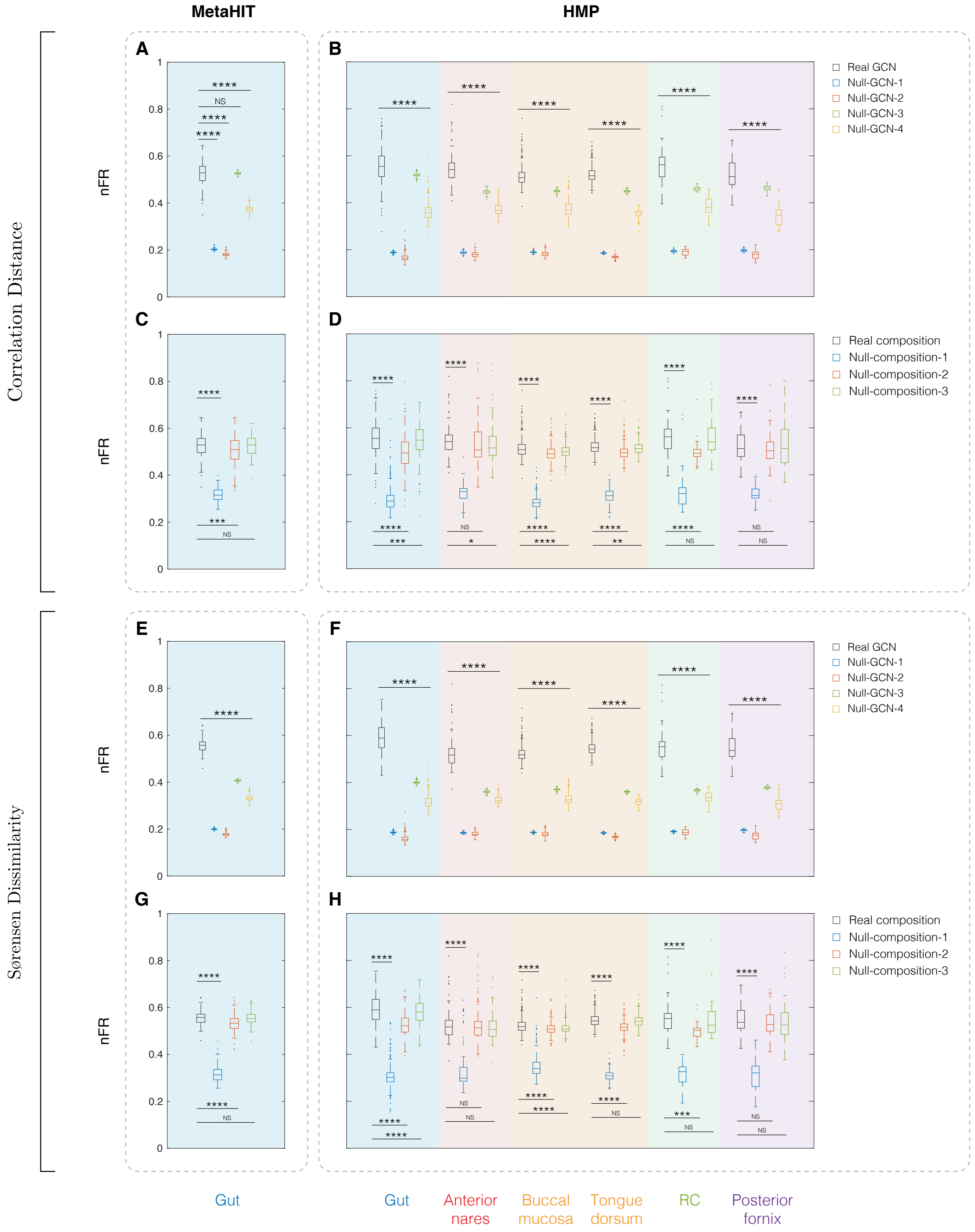

Supplementary Figure 1: Normalized functional redundancy calculated using two different distance measures. (**A-D**), Correlation distance [17]; (**E-H**), Sørensen dissimilarity [18]. Microbiome samples from the MetaHIT [10, 19] for gut (n = 177 samples), as well as the HMP [20, 21, 22] for six different body sites (gut, n = 549 samples; anterior nares n = 87 samples; buccal mucosa n = 368 samples; tongue dorsum, n = 418 samples; retroauricular crease, RC, n = 36 samples; posterior fornix n = 52 samples), were analyzed. (**A-B, E-F**), The box plots of normalized function redundancy are shown for the real GCN (black box), as well as the randomized GCNs (colored boxes) using four different randomization schemes: complete randomization (Null-GCN-1); species-degree preserving randomization (Null-GCN-2); KO-degree preserving randomization (Null-GCN-3); species- and KO-degree preserving randomization (Null-GCN-4). (**C-D, G-H**), The normalized function redundancy is calculated for the real microbial composition (black box), as well as the randomized microbial compositions (colored boxes) using three different randomization schemes: Randomized microbial assemblage generated by randomly choosing the same number of species from the species pool but keeping the species abundance profile unchanged (Null-composition-1); Randomized microbial abundance profiles through random permutation of non-zero abundance for each sample across different species (Null-composition-2); Randomized microbial abundance profiles through random permutation of non-zero abundance for each species across different samples (Null-composition-3). See SI Sec. 3 for the details of the null models. Boxes indicate the interquartile range between the first and third quartiles with the central mark inside each box indicating the median. Whiskers extend to the lowest and highest values within 1.5 times the interquartile range. Statistical analysis was performed using a two-sided Wilcoxon signed rank test. Significance levels: FDR-corrected p-value <0.05 (*), <0.01 (**), <0.001 (***), <0.0001 (****); >0.05 (NS, non-significant). See Source data for the exact FDR-corrected p-values.

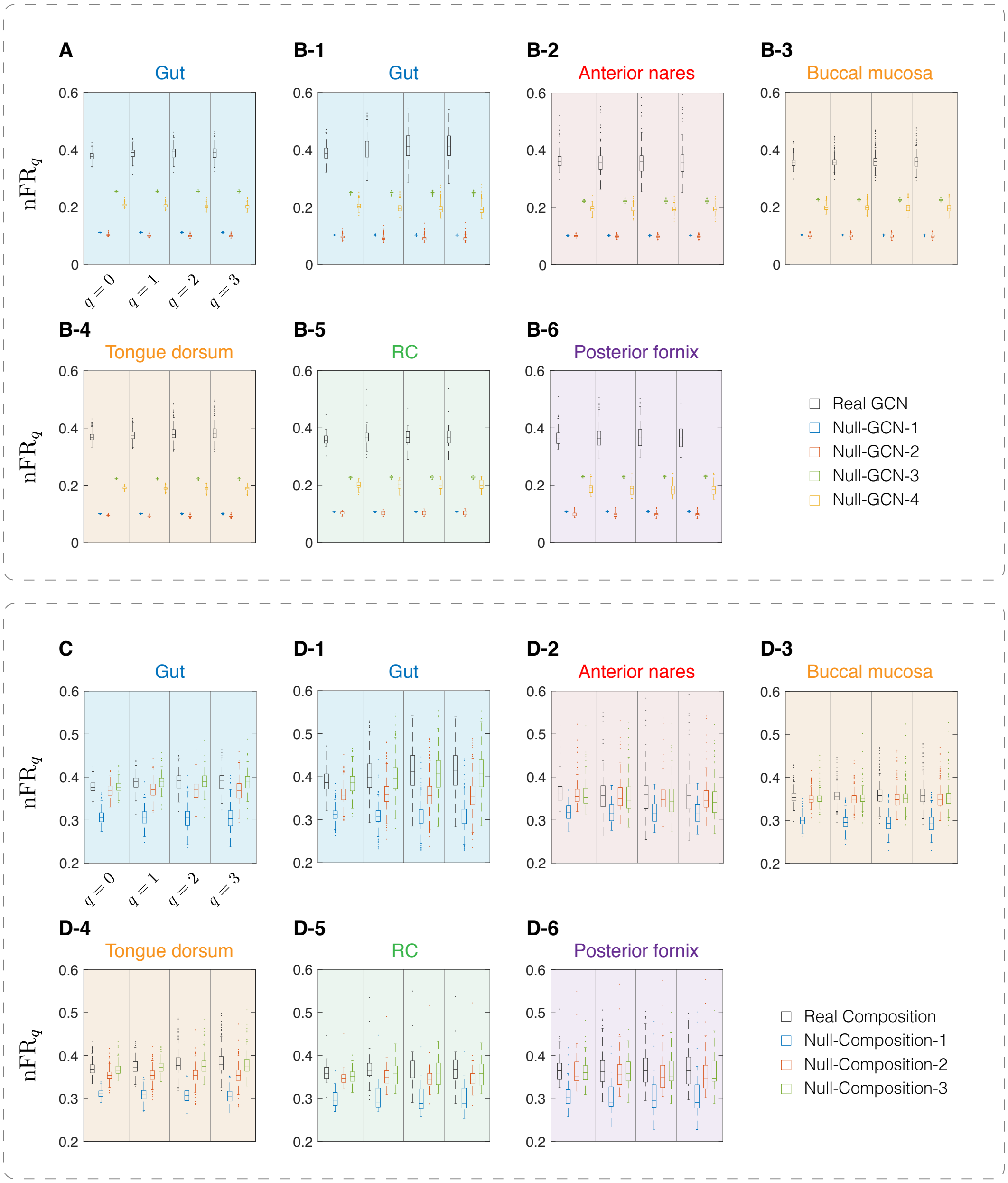
A
Gut
B-1
Gut
B-2
Anterior nares
B-3
Buccal mucosa
B-4
Tongue dorsum
B-5
RC
B-6
Posterior fornix
nFR_q
$q=0$
$q=1$
$q=2$
$q=3$
Real GCN
Null-GCN-1
Null-GCN-2
Null-GCN-3
Null-GCN-4
C
Gut
D-1
Gut
D-2
Anterior nares
D-3
Buccal mucosa
D-4
Tongue dorsum
D-5
RC
D-6
Posterior fornix
Real Composition
Null-Composition-1
Null-Composition-2
Null-Composition-3

Supplementary Figure 2: Hill number based normalized functional redundancy of microbiome samples in different body sites for two metagenomic datasets. (**A, C**), MetaHIT [10, 19] (n = 177 samples); (**B, D**), HMP [20, 21, 22] (for six different body sites: gut, n = 549 samples; anterior nares n = 87 samples; buccal mucosa n = 368 samples; tongue dorsum, n = 418 samples; retroauricular crease, RC, n = 36 samples; posterior fornix n = 52 samples). (**Upper panels**), The box plots of nFR_q were shown for the real GCN (black box), as well as the randomized GCNs (colored boxes) using four different randomization schemes: complete randomization (blue); species-degree preserving randomization (red); KO-degree preserving randomization (green); species- and KO-degree preserving randomization (yellow). (**Lower panels**), The box plots of nFR_q were shown for the real microbial composition (black box), as well as the randomized microbial compositions (colored boxes) using three different randomization schemes: Randomized microbial assemblage generated by randomly choosing the same number of species from the species pool but keeping the species abundance profile unchanged (blue); Randomized microbial abundance profiles through random permutation of non-zero abundance for each sample across different species (red); Randomized microbial abundance profiles through random permutation of non-zero abundance for each species across different samples (green). Boxes indicate the interquartile range between the first and third quartiles with the central mark inside each box indicating the median. Whiskers extend to the lowest and highest values within 1.5 times the interquartile range. See Sec. 1 for the definition of Hill number based functional redundancy.

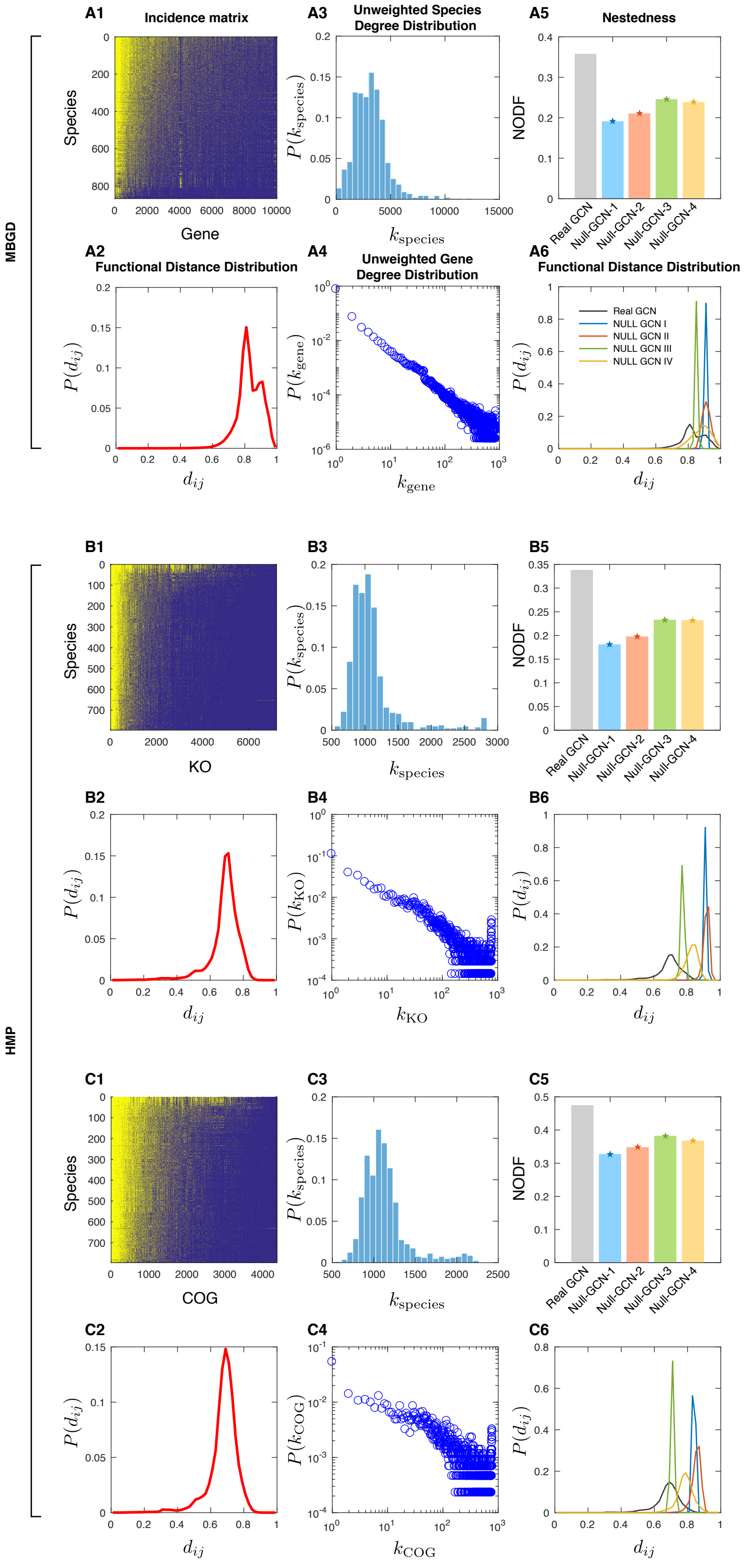
MBGD
A1
Incidence matrix
Species
Gene
A2
Functional Distance Distribution
$P(d_{ij})$
d_{ij}
A3
Unweighted Species Degree Distribution
$P(k_{\text{species}})$
k_{species}
A4
Unweighted Gene Degree Distribution
$P(k_{\text{gene}})$
k_{gene}
A5
Nestedness
NODF
Real GCN
Null-GCN-1
Null-GCN-2
Null-GCN-3
Null-GCN-4
A6
Functional Distance Distribution
Real GCN
NULL GCN I
NULL GCN II
NULL GCN III
NULL GCN IV
$P(d_{ij})$
d_{ij}
HMP
B1
Species
KO
B2
$P(d_{ij})$
d_{ij}
B3
$P(k_{\text{species}})$
k_{species}
B4
$P(k_{\text{KO}})$
k_{KO}
B5
NODF
Real GCN
Null-GCN-1
Null-GCN-2
Null-GCN-3
Null-GCN-4
B6
$P(d_{ij})$
d_{ij}
C1
Species
COG
C2
$P(d_{ij})$
d_{ij}
C3
$P(k_{\text{species}})$
k_{species}
C4
$P(k_{\text{COG}})$
k_{COG}
C5
NODF
Real GCN
Null-GCN-1
Null-GCN-2
Null-GCN-3
Null-GCN-4
C6
$P(d_{ij})$
d_{ij}

Supplementary Figure 3: Structural properties of the genomic content networks (GCNs). **A**, The GCN constructed from the MicroBial Genome Database (MBGD) [23]. **B**, The GCN constructed from the Human Microbiome Project (HMP) reference genomes [7, 20, 21] with function nodes representing KEGG Orthologs (KOs) [24]. **C**, The GCN constructed from the HMP reference genomes with function nodes representing Clusters of Orthologous Groups of proteins (COGs) [25]. (**A-1, B-1, C-1**) The incidence matrix of the GCN, where the presence (or absence) of a link is colored in yellow (or blue), respectively. We organized this matrix using the Nestedness Temperature Calculator (NTC) to emphasize its nested structure [26]. Note that NTC is computationally intensive. Here, for visualization purpose, in (**A-1**) we only show the top 10,000 genes with the highest degree. (**A-2, B-2, C-2**), The distribution of functional distances (d_{ij}) between two different species. The bin size is 0.02. (**A-3, B-3, C-3**), Distribution of the unweighted degrees of species. Here, the unweighted degree of a species is just the number of distinct genes (or KOs, COGs) in its genome. (**A-4, B-4, C-4**), Distribution of the unweighted degrees of genes (or KOs, COGs). Here, the unweighted degree of a gene (or KO, COG) is just the number of species whose genomes contain this gene (or KO, COG). (**A-5, B-5, C-5**), The nestedness based on the NODF measure [27] of the real GCN (gray bar), as well as the randomized GCNs (colored bars) using four different GCN randomization schemes: I, complete randomization (blue); II, species-degree preserving randomization (red); III, Gene- (or KO-, COG-) degree preserving randomization (green); IV, Species- and gene- (or KO-, COG-) degree preserving randomization (yellow). Here the (weighted) gene- (or KO-, COG-) degree is the sum of copy numbers of this gene (or KO, COG) in those genomes that contain it, and the (weighted) species-degree is the sum of copy numbers of those genes (or KOs, COGs) in this species' genome. For each randomization scheme, 100 realizations are generated, and the standard deviation is smaller than the symbol size (pentagram). (**A-6, B-6, C-6**), The distribution of functional distances (d_{ij}) between different species of the real GCN (black lines), compared with the randomized GCNs (colored lines). We generated 100 realizations for each randomization scheme, and the bin size is 0.02. See SI Sec. 3 for details of GCN randomizations.

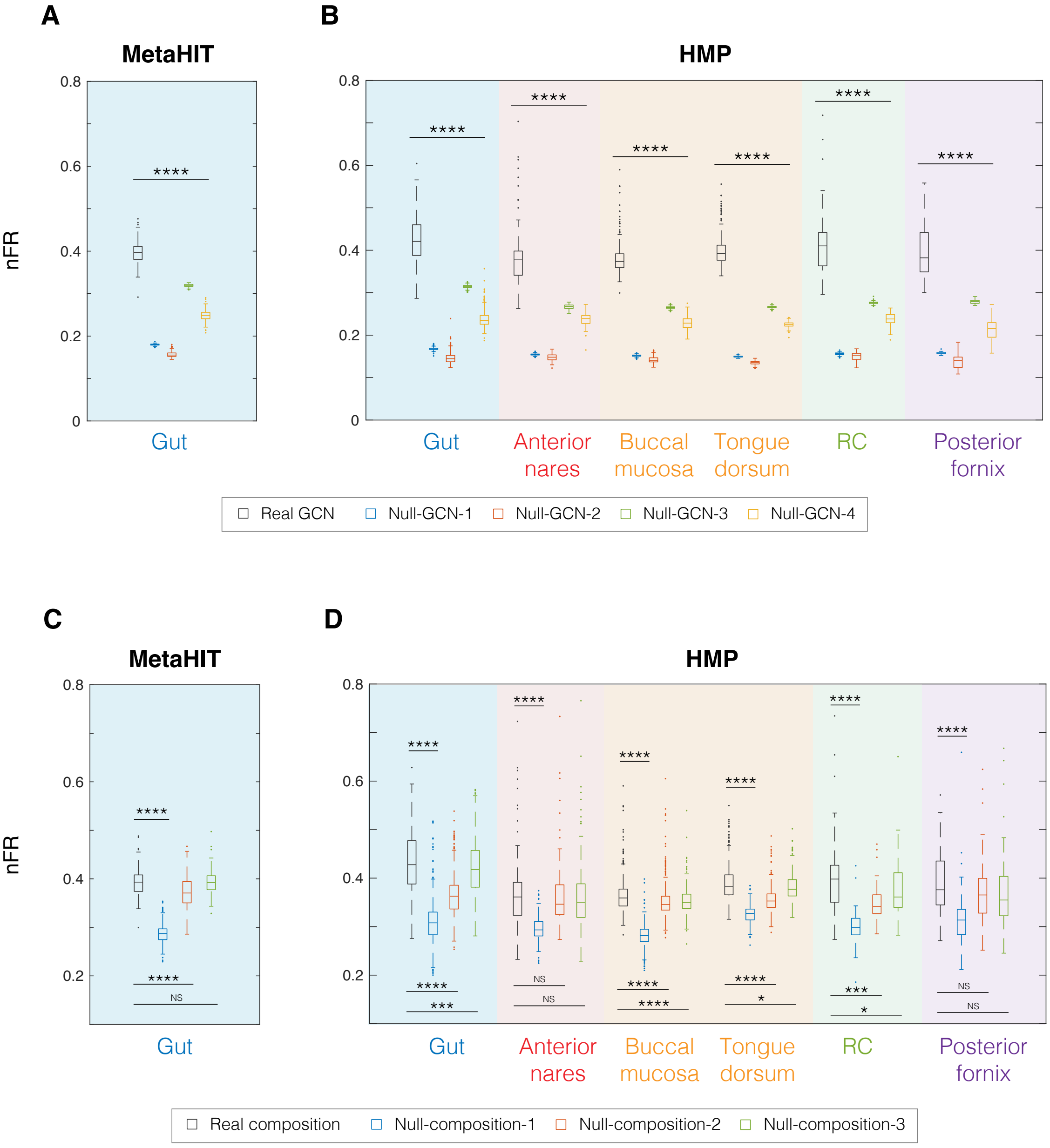
A
MetaHIT
nFR
0.8
0.6
0.4
0.2
0
****
Gut
B
HMP
Gut
Anterior nares
Buccal mucosa
Tongue dorsum
RC
Posterior fornix
Real GCN
Null-GCN-1
Null-GCN-2
Null-GCN-3
Null-GCN-4
C
MetaHIT
D
HMP
NS
Real composition
Null-composition-1
Null-composition-2
Null-composition-3

Supplementary Figure 4: Normalized functional redundancy calculated from body site-specific GCNs with COG annotation. Microbiome samples from the MetaHIT [10, 19] (for gut, $n = 177$ samples), as well as HMP [20, 21, 22] (for six different body sites: gut, $n = 549$ samples; anterior nares $n = 87$ samples; buccal mucosa $n = 368$ samples; tongue dorsum, $n = 418$ samples; retroauricular crease, RC, $n = 36$ samples; posterior fornix $n = 52$ samples), were analyzed. (**A-B**), The box plots of normalized function redundancy are shown for the real GCN (black box), as well as the randomized GCNs (colored boxes) using four different randomization schemes: complete randomization (Null-GCN-1); species-degree preserving randomization (Null-GCN-2); COG-degree preserving randomization (Null-GCN-3); species- and COG-degree preserving randomization (Null-GCN-4). (**C-D**), The normalized function redundancy is calculated for the real microbial composition (black box), as well as the randomized microbial compositions (colored boxes) using three different randomization schemes: Randomized microbial assemblage generated by randomly choosing the same number of species from the species pool but keeping the species abundance profile unchanged (Null-composition-1); Randomized microbial abundance profiles through random permutation of non-zero abundance for each sample across different species (Null-composition-2); Randomized microbial abundance profiles through random permutation of non-zero abundance for each species across different samples (Null-composition-3). Boxes indicate the interquartile range between the first and third quartiles with the central mark inside each box indicating the median. Whiskers extend to the lowest and highest values within 1.5 times the interquartile range. Statistical analysis was performed using a two-sided Wilcoxon signed rank test. Significance levels: FDR-corrected p-value<0.05 (*), <0.01 (**), <0.001 (***), <0.0001 (****); >0.05 (NS, non-significant). See Source data for the exact FDR-corrected p-values.

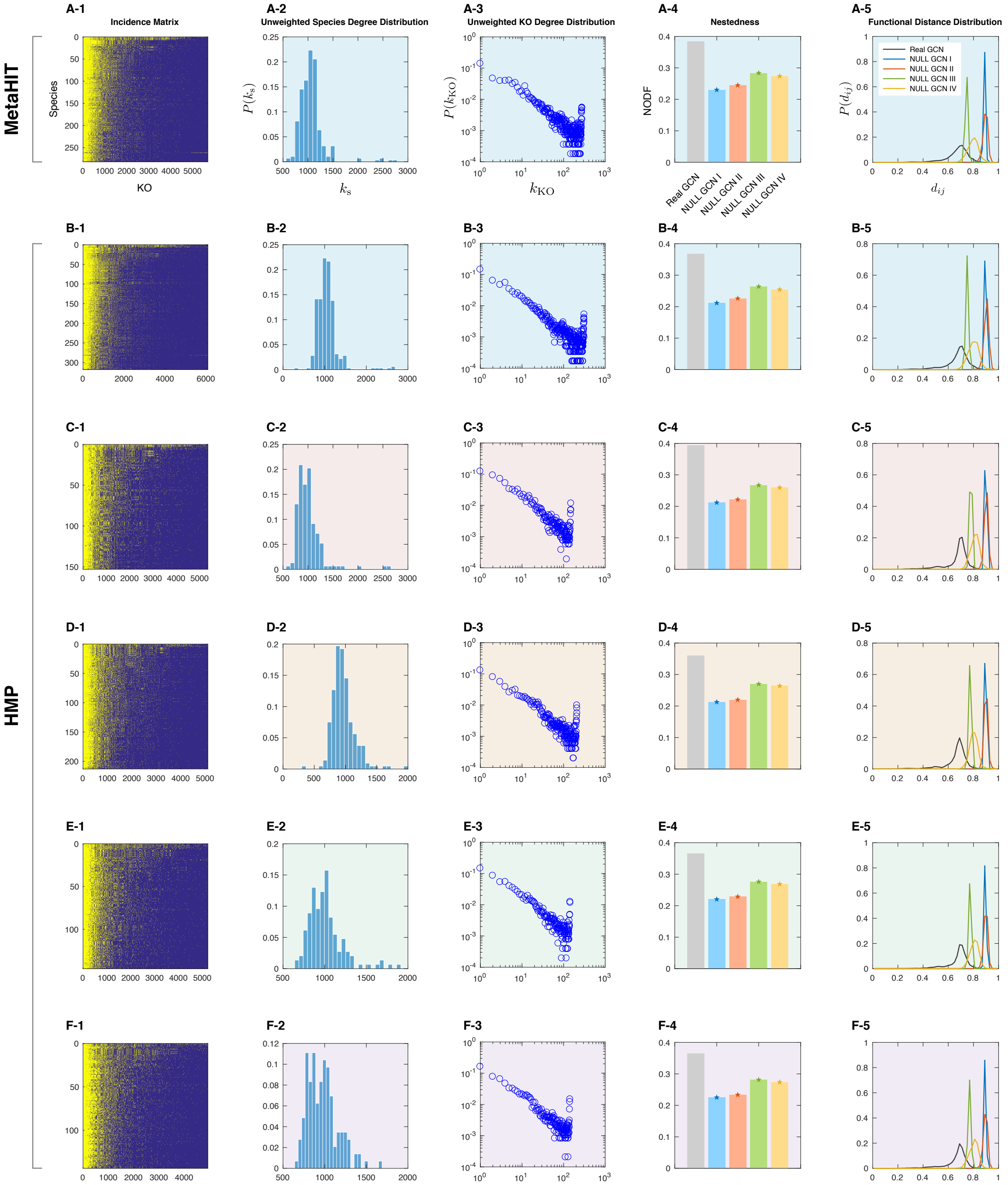
A-1
Incidence Matrix
MetaHIT
Species
KO
A-2
Unweighted Species Degree Distribution
A-3
Unweighted KO Degree Distribution
A-4
Nestedness
NODF
Real GCN
NULL GCN I
NULL GCN II
NULL GCN III
NULL GCN IV
A-5
Functional Distance Distribution
HMP
B-1
B-2
B-3
B-4
B-5
C-1
C-2
C-3
C-4
C-5
D-1
D-2
D-3
D-4
D-5
E-1
E-2
E-3
E-4
E-5
F-1
F-2
F-3
F-4
F-5

Supplementary Figure 5: Structural properties of the body site-specific genomic content networks (GCNs) constructed from two metagenomic datasets: MetaHIT [10, 19] and HMP [20, 21, 22]. **A**, MetaHIT, gut (stool); **B**, HMP, gut (stool); **C**, HMP, airway (anterior nares); **D**, HMP, oral (buccal mucosa); **E**, HMP, skin (retroauricular crease); **F**, HMP, vaginal (posterior fornix). (**column-1**), The incidence matrix of the GCN shown at the species-KO level, where the presence (or absence) of a link between a species and a KO is colored in yellow (or blue), respectively. We organized this matrix using the Nestedness Temperature Calculator to emphasize its nested structure. (**column-2**), The unweighted species degree distribution. (**column-3**), The unweighted KO degree distribution. (**column-4**), The nestedness based on the NODF measure of the real GCN (gray bar), as well as the randomized GCNs (colored bars) using four different GCN randomization schemes: I, complete randomization (blue); II, Species-degree preserving randomization (red); III, KO-degree preserving randomization (green); IV, Species- and KO-degree preserving randomization (yellow). For each randomization scheme, 100 realizations are generated, and the standard deviation is smaller than the symbol size (pentagram). See SI Sec. 3 for details. (**column-5**), The distribution of functional distances (d_{ij}) between different species of the real GCN (black lines), compared with the randomized GCNs (colored lines) using the same GCN randomization schemes as in Column-4. We generated 100 realizations for each randomization scheme, and the bin size is 0.02.

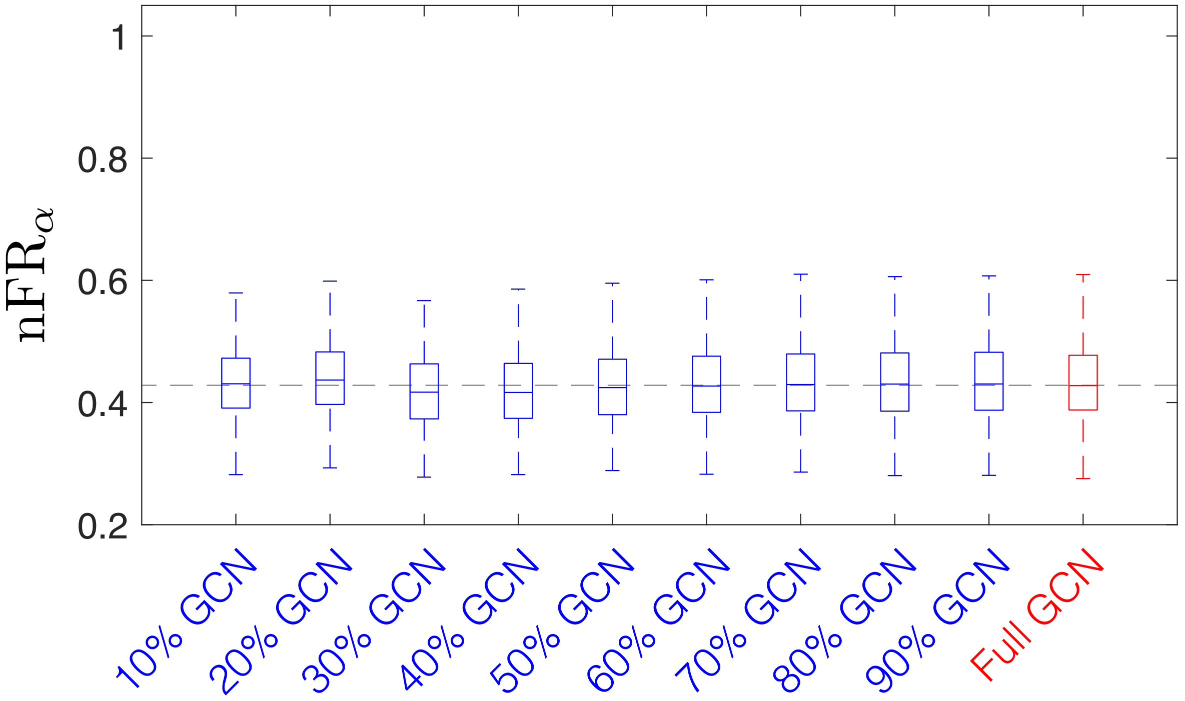

Supplementary Figure 6: Normalized functional redundancy as a function of the integrity of the GCN. Here we analyze SMS data of stool microbiome samples from HMP [20, 21, 22] ($n = 549$). From right to the left, KOs are gradually and randomly removed from the GCN. Red box represents the result of the original or full GCN. 90% GCN means that 10% of the KOs have been randomly removed. 10% GCN means that 90% of the KOs have been randomly removed. Boxes indicate the interquartile range between the first and third quartiles with the central mark inside each box indicating the median. Whiskers extend to the lowest and highest values within 1.5 times the interquartile range.

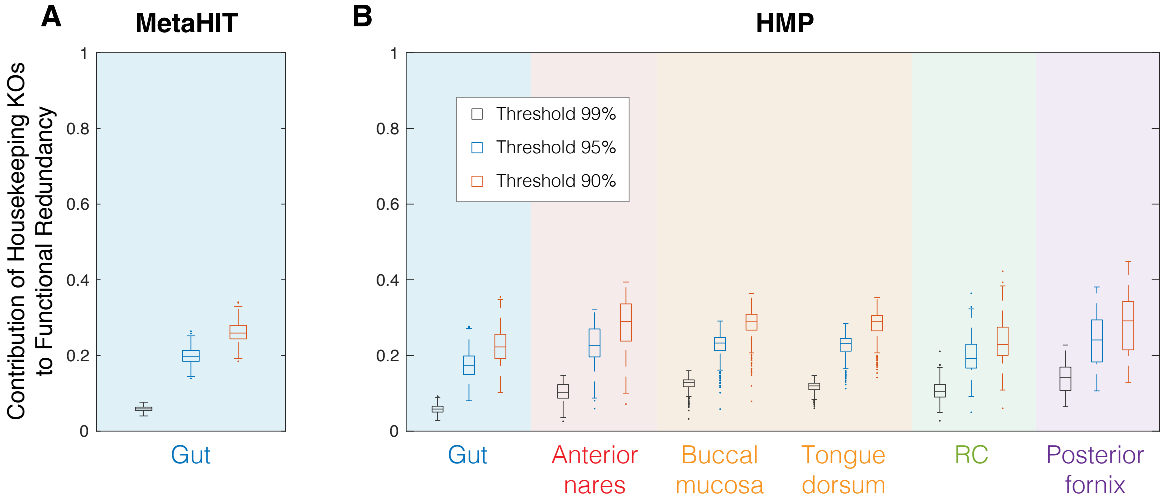

Supplementary Figure 7: Contribution of housekeeping genes to the functional redundancy of microbiome samples. Here we analyze microbiome samples from different body sites for two metagenomic datasets: (**A**) MetaHIT [10, 19] (for gut, $n = 177$ samples); (**B**) HMP [20, 21, 22] (for six different body sites: gut, $n = 549$ samples; anterior nares $n = 87$ samples; buccal mucosa $n = 368$ samples; tongue dorsum, $n = 418$ samples; retroauricular crease, RC, $n = 36$ samples; posterior fornix $n = 52$ samples). For each bodysite, the housekeeping KOs are considered as the KOs that are present in a large fraction of the existing genomes. Here we used three fraction thresholds: 99%, 95%, and 90% to define housekeeping KOs. Denote the functional redundancy calculated from the real GCN as FR, and the functional redundancy calculated from the real GCN with housekeeping KOs removed as FR_{woKO}. Then the contribution of housekeeping KOs to the functional redundancy of a microbiome sample, denotes as FR_{KO}, can be quantified as $\text{FR}_{\text{KO}} = 1 - \text{FR}_{\text{woKO}}/\text{FR}$. Apparently, if we relax the fraction threshold from 99% to 90%, FR_{KO} will increase. But even with the threshold 90% (i.e., those housekeeping KOs appear in the genomes of 90% species), FR_{KO} is still generally smaller than 30%. This indicates that the housekeeping KOs only contribute a small part of the functional redundancy observed in human microbiome samples. Boxes indicate the interquartile range between the first and third quartiles with the central mark inside each box indicating the median. Whiskers extend to the lowest and highest values within 1.5 times the interquartile range.

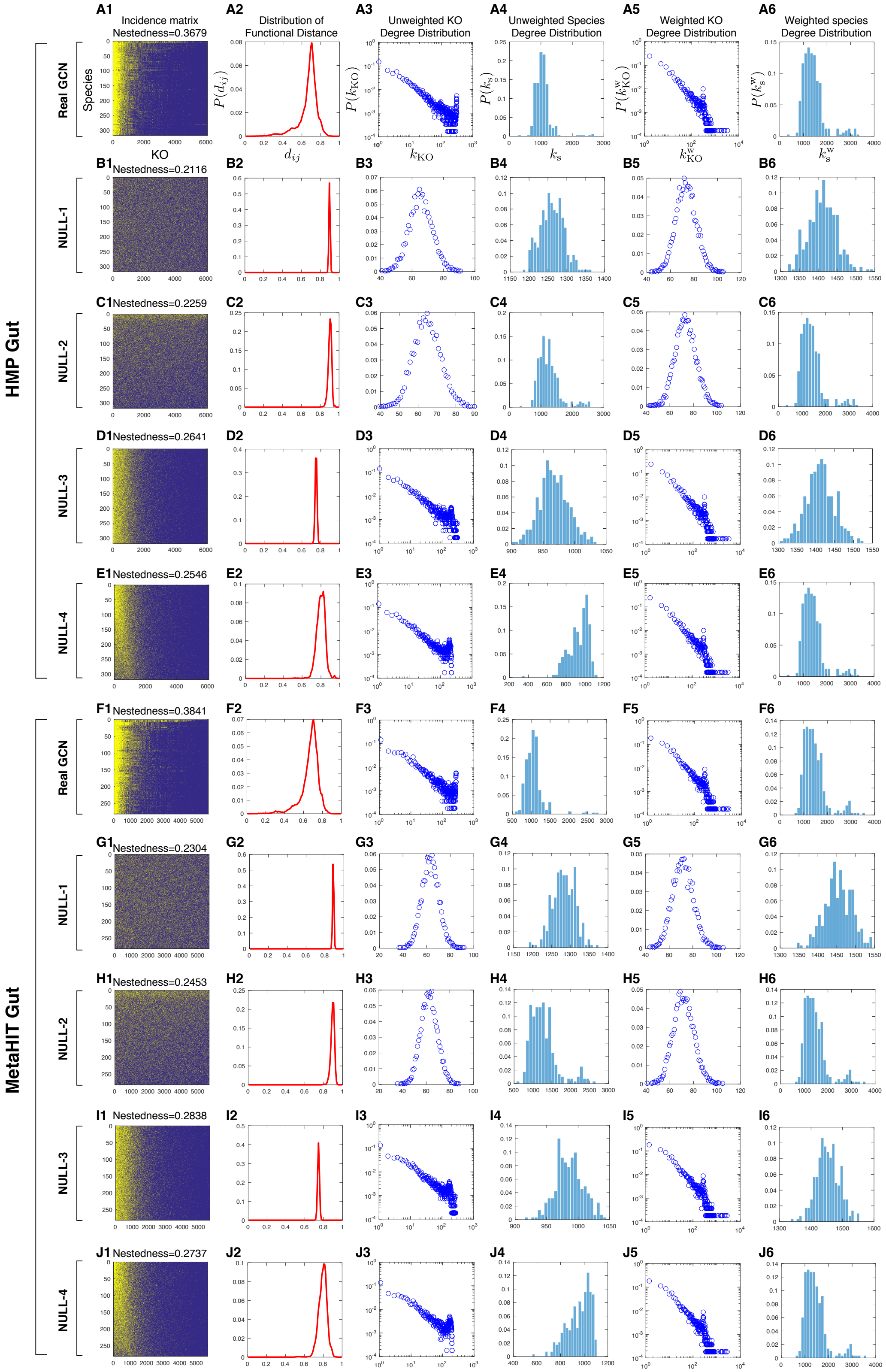

A1
Incidence matrix
Nestedness=0.3679
A2
Distribution of
Functional Distance
A3
Unweighted KO
Degree Distribution
A4
Unweighted Species
Degree Distribution
A5
Weighted KO
Degree Distribution
A6
Weighted species
Degree Distribution
Species
KO
$P(d_{ij})$
d_{ij}
$P(k_{KO})$
k_{KO}
$P(k_s)$
k_s
$P(k^w_{KO})$
k^w_{KO}
$P(k^w_s)$
k^w_s
HMP Gut
Real GCN
B1 Nestedness=0.2116
NULL-1
C1 Nestedness=0.2259
NULL-2
D1 Nestedness=0.2641
NULL-3
E1 Nestedness=0.2546
NULL-4
MetaHIT Gut
Real GCN
F1 Nestedness=0.3841
G1 Nestedness=0.2304
NULL-1
H1 Nestedness=0.2453
NULL-2
I1 Nestedness=0.2838
NULL-3
J1 Nestedness=0.2737
NULL-4
B2
B3
B4
B5
B6
C2
C3
C4
C5
C6
D2
D3
D4
D5
D6
E2
E3
E4
E5
E6
F2
F3
F4
F5
F6
G2
G3
G4
G5
G6
H2
H3
H4
H5
H6
I2
I3
I4
I5
I6
J2
J3
J4
J5
J6

Supplementary Figure 8: Structural properties of the gut-GCN constructed from two metagenomic datasets. (**A-E**), HMP [20, 21, 22]. (**F-J**), MetaHIT [10, 19]. (**colomn-1**), The incidence matrix of the GCN shown at the species-KO level, where the presence (or absence) of a link between a species and a KO is colored in yellow (or blue), respectively. We organized this matrix using the Nestedness Temperature Calculator to emphasize its nested structure [26]. The nestedness of this network is calculated based on the NODF measure [27]. (**colomn-2**), The distribution of functional distances (d_{ij}) between different species. (**column-3**), The unweighted KO degree distribution. (**column-4**), The unweighted species degree distribution. (**column-5**), The weighted KO degree distribution, where the (weighted) degree of a KO is the sum of copy numbers of this KO in those species whose genomes contain this KO. (**column-6**), The weighted species degree distribution, where the (weighted) degree of a species is the sum of copy numbers of those KOs in this species' genome. (**A,F**), The structural properties of the real GCN. (**B,G**), Null-1: Completely randomized GCN. (**D,H**) Null-2: Randomized GCN with species-degree preserved. (**D,I**) Null-3: Randomized GCN with KO-degree preserved. (**E,J**) Null-4: Randomized GCN with both species-degree and KO-degree preserved. See SI Sec. 3 for the details of the four different null models.

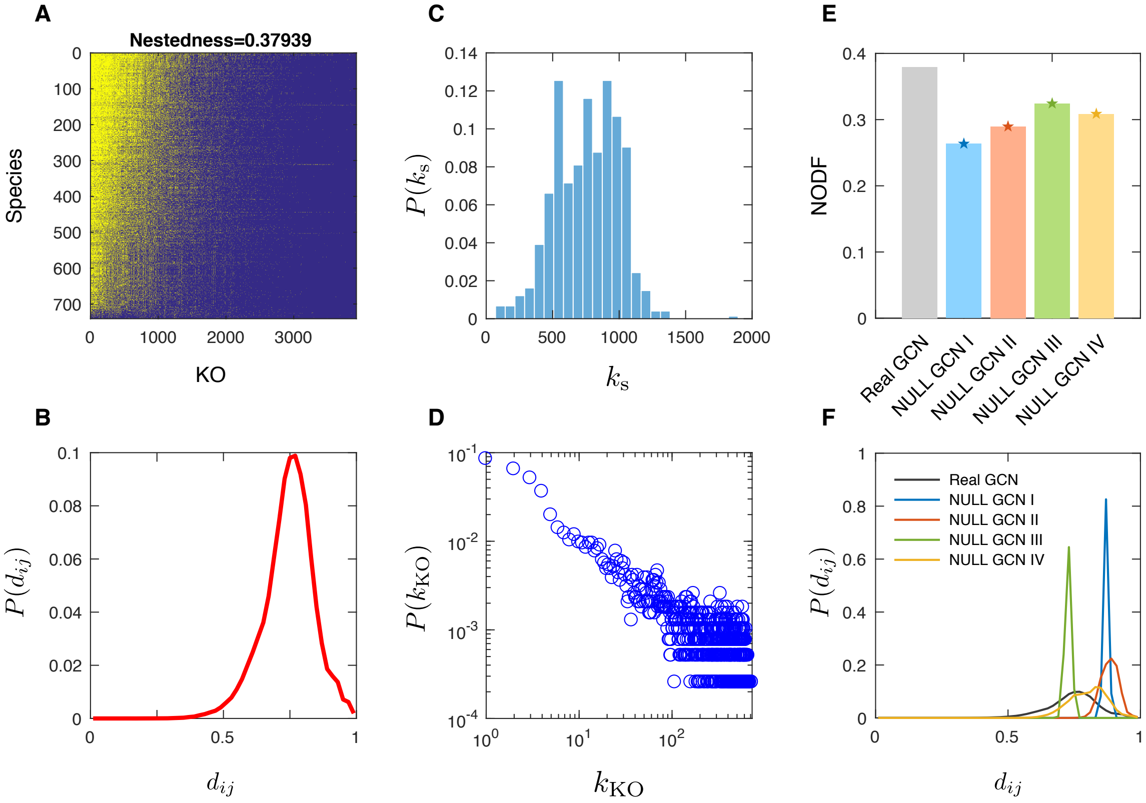

Supplementary Figure 9: Structural properties of the genomic content network (GCN) constructed by *de novo* taxonomic profiling of SMS data. The GCN is constructed for the gut microbiome samples from the MetaHIT dataset [10, 19]. (**A**) The incidence matrix of the GCN, where the presence (or absence) of a species-KO link is colored in yellow (or blue), respectively. We organized this matrix using the Nestedness Temperature Calculator (NTC) to emphasize its nested structure [26]. (**B**), The distribution of functional distances (d_{ij}) between two different species. The bin size is 0.02. (**C**), Distribution of the unweighted degrees of species. (**D**), Distribution of the unweighted degrees of KOs. (**E**), The nestedness based on the NODF measure [27] of the real GCN (gray bar), as well as the randomized GCNs (colored bars) using four different GCN randomization schemes: I, complete randomization (blue); II, species-degree preserving randomization (red); III, KO-degree preserving randomization (green); IV, KO-degree preserving randomization (yellow). For each randomization scheme, 100 realizations are generated, and the standard deviation is smaller than the symbol size (pentagram). (**F**), The distribution of functional distances (d_{ij}) between different species of the real GCN (black lines), compared with the randomized GCNs (colored lines). We generated 100 realizations for each randomization scheme, and the bin size is 0.02. See SI Sec. 3 for details of GCN randomizations. See SI Sec. 2.2 for details of the GCN construction through *de novo* taxonomic profiling.

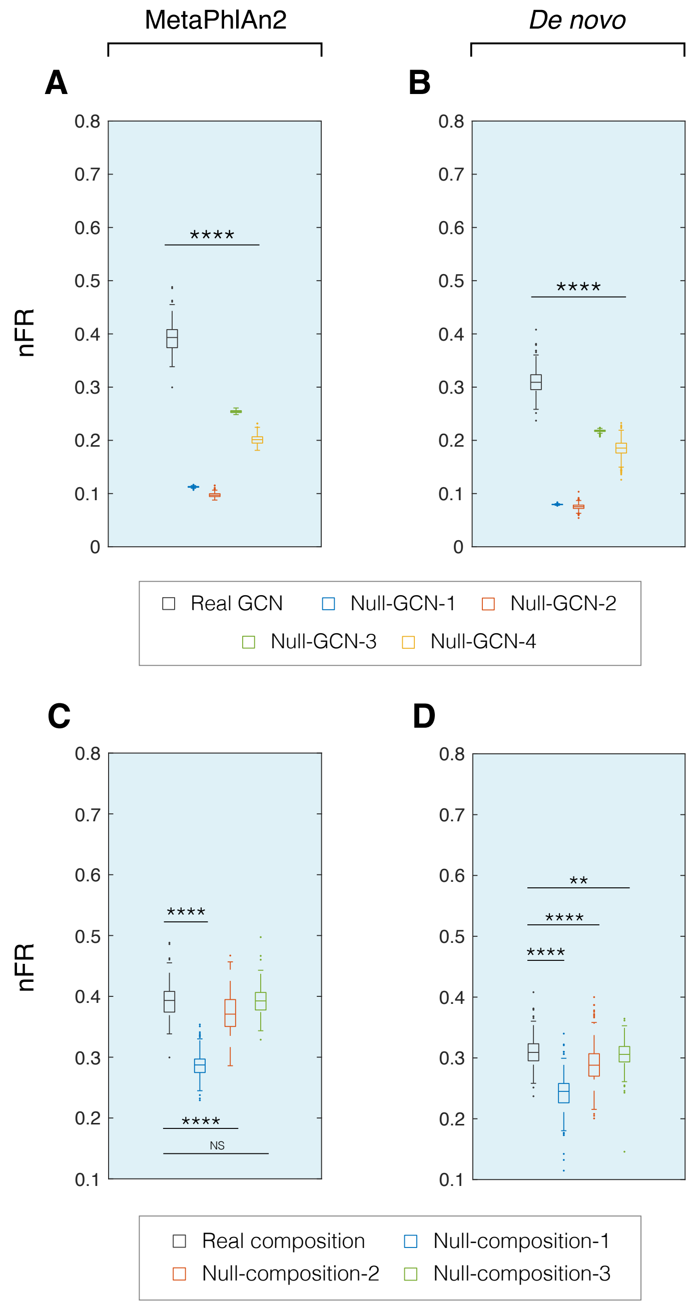
MetaPhlAn2
De novo
A
B
nFR
****
****
Real GCN
Null-GCN-1
Null-GCN-2
Null-GCN-3
Null-GCN-4
C
D
****
****
NS
**
****
****
Real composition
Null-composition-1
Null-composition-2
Null-composition-3

Supplementary Figure 10: Normalized functional redundancy calculated with two different pipelines of taxonomic profiling for metagenomic samples. (**A, C**) MetaPhlAn2 (metagenomic phylogenetic analysis v2.0) using unique clade-specific marker genes [8]; (**B, D**) *De novo* segregation of complex metagenomic data based on binning co-abundant genes across metagenomic samples [10]. Gut microbiome samples from MetaHIT [10, 19] (for gut, $n = 177$ samples) were analyzed. (**A-B**), The box plots of normalized function redundancy are shown for the real GCN (black box), as well as the randomized GCNs (colored boxes) using four different randomization schemes: complete randomization (Null-GCN-1); species-degree preserving randomization (Null-GCN-2); KO-degree preserving randomization (Null-GCN-3); species- and KO-degree preserving randomization (Null-GCN-4). (**C-D**), The normalized function redundancy is calculated for the real microbial composition (black box), as well as the randomized microbial compositions (colored boxes) using three different randomization schemes: Randomized microbial assemblage generated by randomly choosing the same number of species from the species pool but keeping the species abundance profile unchanged (Null-composition-1); Randomized microbial abundance profiles through random permutation of non-zero abundance for each sample across different species (Null-composition-2); Randomized microbial abundance profiles through random permutation of non-zero abundance for each species across different samples (Null-composition-3). Boxes indicate the interquartile range between the first and third quartiles with the central mark inside each box indicating the median. Whiskers extend to the lowest and highest values within 1.5 times the interquartile range. Statistical analysis was performed using a two-sided Wilcoxon signed rank test. Significance levels: FDR-corrected p-value<0.05 (*), <0.01 (**), <0.001 (***), <0.0001 (****); >0.05 (NS, non-significant). See Source data for the exact FDR-corrected p-values.

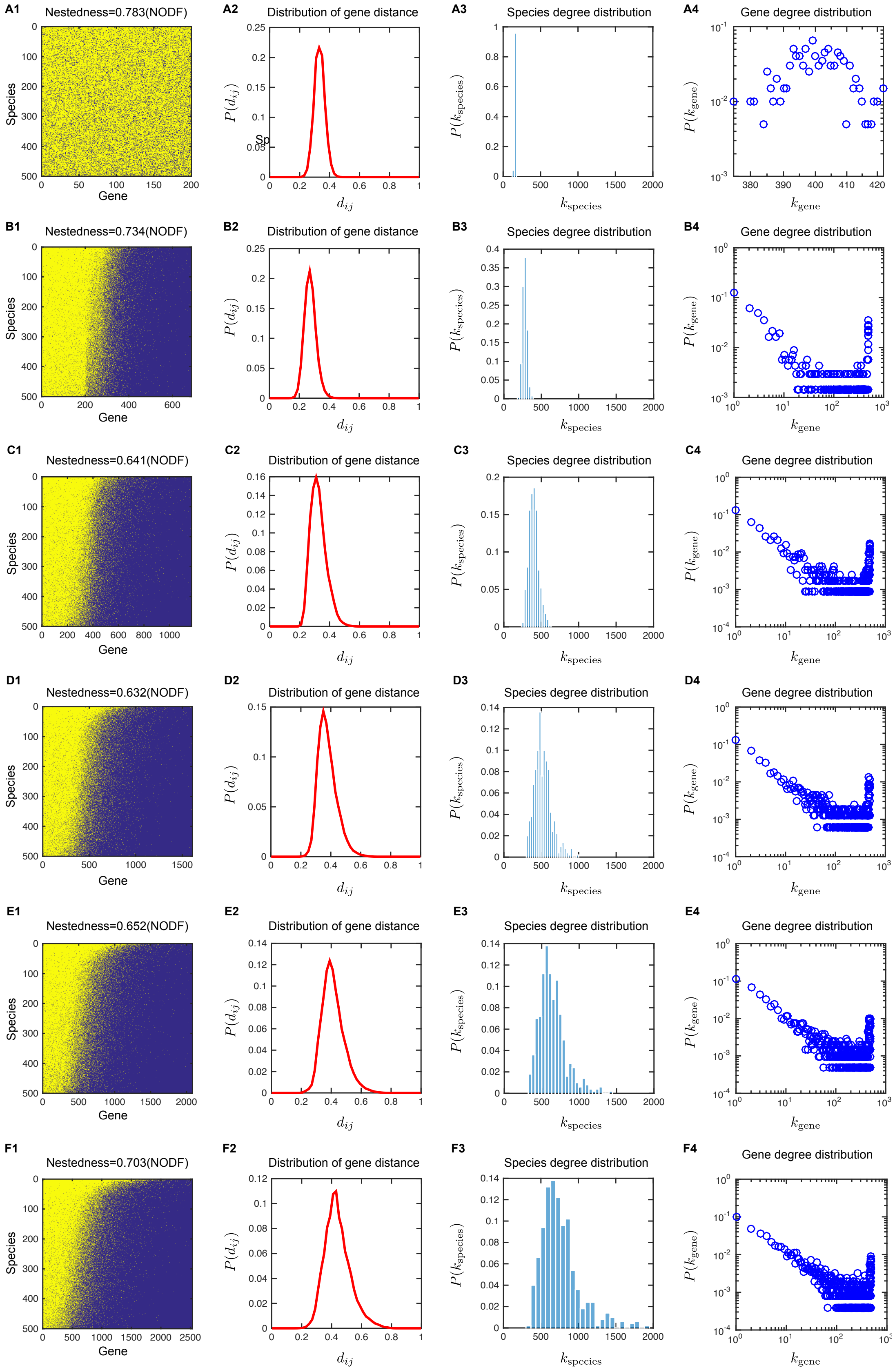
A1
Nestedness=0.783(NODF)
Species
Gene
A2
Distribution of gene distance
$P(d_{ij})$
d_{ij}
A3
Species degree distribution
$P(k_{\text{species}})$
k_{species}
A4
Gene degree distribution
$P(k_{\text{gene}})$
k_{gene}
B1
Nestedness=0.734(NODF)
Species
Gene
B2
Distribution of gene distance
$P(d_{ij})$
d_{ij}
B3
Species degree distribution
$P(k_{\text{species}})$
k_{species}
B4
Gene degree distribution
$P(k_{\text{gene}})$
k_{gene}
C1
Nestedness=0.641(NODF)
Species
Gene
C2
Distribution of gene distance
$P(d_{ij})$
d_{ij}
C3
Species degree distribution
$P(k_{\text{species}})$
k_{species}
C4
Gene degree distribution
$P(k_{\text{gene}})$
k_{gene}
D1
Nestedness=0.632(NODF)
Species
Gene
D2
Distribution of gene distance
$P(d_{ij})$
d_{ij}
D3
Species degree distribution
$P(k_{\text{species}})$
k_{species}
D4
Gene degree distribution
$P(k_{\text{gene}})$
k_{gene}
E1
Nestedness=0.652(NODF)
Species
Gene
E2
Distribution of gene distance
$P(d_{ij})$
d_{ij}
E3
Species degree distribution
$P(k_{\text{species}})$
k_{species}
E4
Gene degree distribution
$P(k_{\text{gene}})$
k_{gene}
F1
Nestedness=0.703(NODF)
Species
Gene
F2
Distribution of gene distance
$P(d_{ij})$
d_{ij}
F3
Species degree distribution
$P(k_{\text{species}})$
k_{species}
F4
Gene degree distribution
$P(k_{\text{gene}})$
k_{gene}

Supplementary Figure 11: The genome evolution model can evolve into a steady state where all the key topological features of the GCN are relatively stable. $q_{\text{hgt}} = 0.795$, $q_{\text{gg}} = 0.005$, $q_{\text{gl}} = 0.2$ and $h = 2$. The initial network consists of 500 species and 200 genes. Topological features at different time steps (**A1-A4**), $t = 0$. (**B1-B4**), $t = 1 \times 10^5$. (**C1-C4**), $t = 2 \times 10^5$. (**D1-D4**), $t = 3 \times 10^5$. (**E1-E4**), $t = 4 \times 10^5$. (**F1-F4**), $t = 5 \times 10^5$.

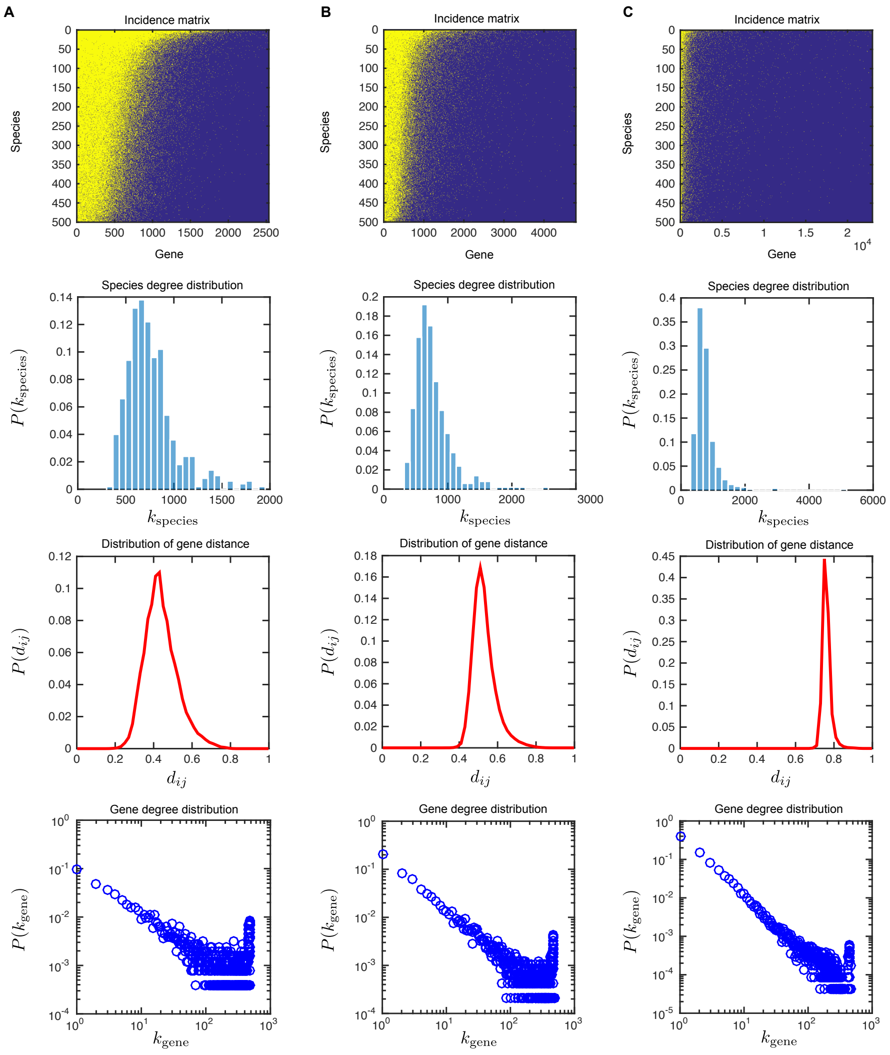

Supplementary Figure 12: Key topological features of the GCN simulated from the genome evolution model with different gene gain rates. The initial GCN consists of 500 species and 200 genes. $h = 2$. $q_{\mathrm{gl}} = 0.2$ (**A**), $q_{\mathrm{hgt}} = 0.795$, $q_{\mathrm{gg}} = 0.005$. (**B**), $q_{\mathrm{hgt}} = 0.79$, $q_{\mathrm{gg}} = 0.01$. (**C**), $q_{\mathrm{hgt}} = 0.75$, $q_{\mathrm{gg}} = 0.05$.

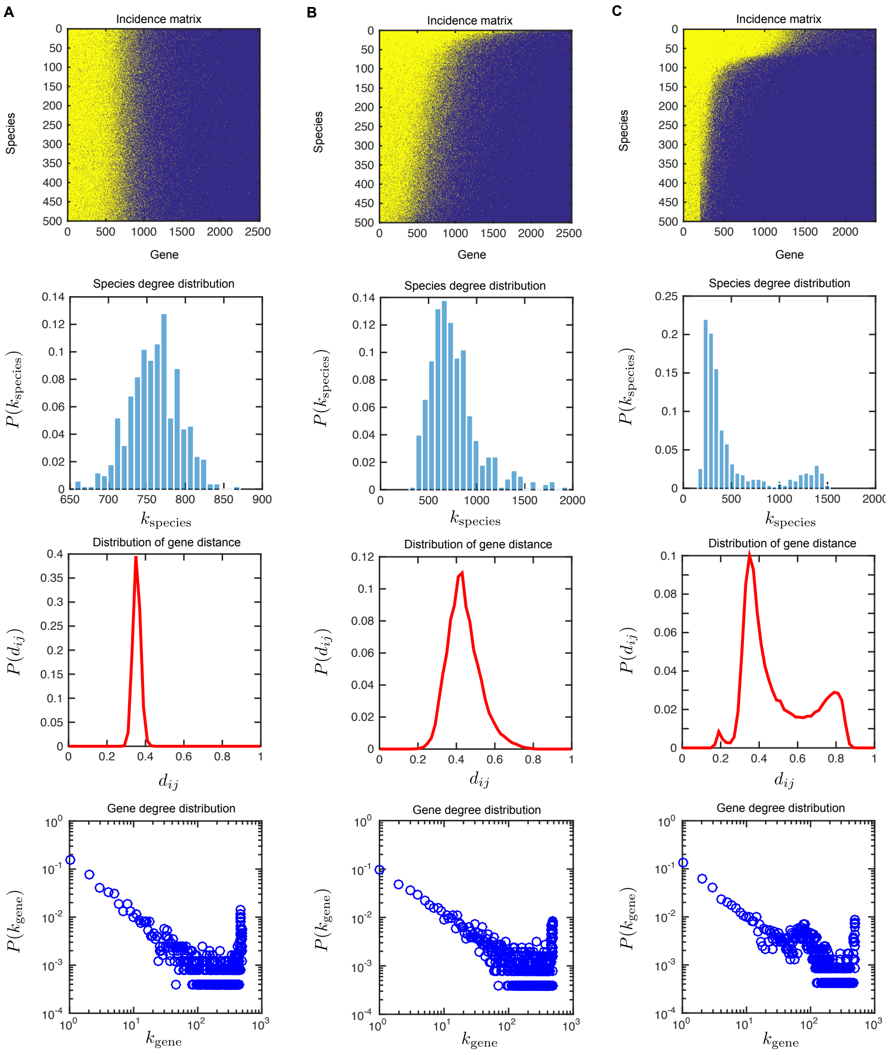

Supplementary Figure 13: Key topological features of the GCN simulated from the genome evolution model with different selection pressure. The initial GCN consists of 500 species and 200 genes. $q_{\rm hgt} = 0.795$, $q_{\rm gg} = 0.005$, $q_{\rm gl} = 0.2$. (**A**), $h = 0$. (**B**), $h = 2$. (**C**), $h = 4$. Note that $h = 0$ corresponds to the neutral model.

References

[S1] Hill, M. O. Diversity and evenness: A unifying notation and its consequences. *Ecology* **54**, 427–432 (1973).

[S2] Chiu, C.-H. & Chao, A. Distance-based functional diversity measures and their decomposition: A framework based on hill numbers. *PLOS ONE* **9**, e100014 (2014).

[S3] Rao, C. R. Diversity and dissimilarity coefficients: A unified approach. *Theoretical Population Biology* **21**, 24–43 (1982).

[S4] de Bello, F. *et al.* Importance of species abundance for assessment of trait composition: an example based on pollinator communities. *Community Ecology* **8**, 163–170 (2007).

[S5] Pillar, V. D. *et al.* Functional redundancy and stability in plant communities. *Journal of Vegetation Science* **24**, 963–974 (2013).

[S6] Kang, S. *et al.* Functional redundancy instead of species redundancy determines community stability in a typical steppe of inner mongolia. *PLoS ONE* **10**, 1–11 (2015).

[S7] Markowitz, V. M. *et al.* Img: the integrated microbial genomes database and comparative analysis system. *Nucleic Acids Research* **40**, D115–D122 (2011).

[S8] Truong, D. T. *et al.* Metaphlan2 for enhanced metagenomic taxonomic profiling. *Nature methods* **12**, 902 (2015).

[S9] Segata, N. *et al.* Metagenomic microbial community profiling using unique clade-specific marker genes. *Nature methods* **9**, 811 (2012).

[S10] Nielsen, H. B. *et al.* Identification and assembly of genomes and genetic elements in complex metagenomic samples without using reference genomes. *Nature biotechnology* **32**, 822 (2014).

[S11] Kultima, J. R. *et al.* Mocat: a metagenomics assembly and gene prediction toolkit. *PloS one* **7**, e47656 (2012).

[S12] Li, R. *et al.* De novo assembly of human genomes with massively parallel short read sequencing. *Genome research* **20**, 265–272 (2010).

[S13] Zhu, W., Lomsadze, A. & Borodovsky, M. Ab initio gene identification in metagenomic sequences. *Nucleic acids research* **38**, e132–e132 (2010).

[S14] Kent, W. J. Blat–the blast-like alignment tool. *Genome research* **12**, 656–664 (2002).

[S15] Li, R. *et al.* Soap2: an improved ultrafast tool for short read alignment. *Bioinformatics* **25**, 1966–1967 (2009).

[S16] McCallum, A., Nigam, K. & Ungar, L. H. Efficient clustering of high-dimensional data sets with application to reference matching. In *Proceedings of the sixth ACM SIGKDD international conference on Knowledge discovery and data mining*, 169–178 (ACM, 2000).

[S17] McCune, B., Grace, J. B. & Urban, D. L. *Analysis of ecological communities*, vol. 28 (MjM software design Gleneden Beach, OR, 2002).

[S18] Sørensen, T. A method of establishing groups of equal amplitude in plant sociology based on similarity of species and its application to analyses of the vegetation on danish commons. *Biol. Skr.* **5**, 1–34 (1948).

[S19] Qin, J. *et al.* A human gut microbial gene catalogue established by metagenomic sequencing. *Nature* **464**, 59 (2010).

[S20] The Human Microbiome Project Consortium. Structure, function and diversity of the healthy human microbiome. *Nature* **486**, 207 (2012).

[S21] Methé, B. A. *et al.* A framework for human microbiome research. *Nature* **486**, 215 (2012).

[S22] Lloyd-Price, J. *et al.* Strains, functions and dynamics in the expanded human microbiome project. *Nature* **550**, 61 (2017).

[S23] Uchiyama, I., Higuchi, T. & Kawai, M. Mbgd update 2010: toward a comprehensive resource for exploring microbial genome diversity. *Nucleic Acids Research* **38**, D361–D365 (2009).

[S24] Kanehisa, M. & Goto, S. Kegg: kyoto encyclopedia of genes and genomes. *Nucleic acids research* **28**, 27–30 (2000).

[S25] Tatusov, R. L., Koonin, E. V. & Lipman, D. J. A genomic perspective on protein families. *Science* **278**, 631–637 (1997).

[S26] Atmar, W. & Patterson, B. D. The measure of order and disorder in the distribution of species in fragmented habitat. *Oecologia* **96**, 373–382 (1993).

[S27] Almeida-Neto, M., Guimarães, P., Guimarães, P. R., Loyola, R. D. & Ulrich, W. A consistent metric for nestedness analysis in ecological systems: reconciling concept and measurement. *Oikos* **117**, 1227–1239 (2008).
