# Supplementary material for: Deciphering functional redundancy in the human microbiome
Source: Nat Commun. 2020 Dec 4;11:6217. doi: 10.1038/s41467-020-19940-1 (PMC7719190; doi:10.1038/s41467-020-19940-1)
Supplement: Supplementary file 3 — Reporting Summary [file 41467_2020_19940_MOESM3_ESM.pdf]

nature research

Corresponding author(s): Yang-Yu Liu

Last updated by author(s): Nov 2, 2020

Reporting Summary

Nature Research wishes to improve the reproducibility of the work that we publish. This form provides structure for consistency and transparency in reporting. For further information on Nature Research policies, see our Editorial Policies and the Editorial Policy Checklist.

Statistics

For all statistical analyses, confirm that the following items are present in the figure legend, table legend, main text, or Methods section.

n/a	Confirmed	
☐	☒	The exact sample size (n) for each experimental group/condition, given as a discrete number and unit of measurement
☐	☒	A statement on whether measurements were taken from distinct samples or whether the same sample was measured repeatedly
☐	☒	The statistical test(s) used AND whether they are one- or two-sided<br>*Only common tests should be described solely by name; describe more complex techniques in the Methods section.*
☒	☐	A description of all covariates tested
☒	☐	A description of any assumptions or corrections, such as tests of normality and adjustment for multiple comparisons
☐	☒	A full description of the statistical parameters including central tendency (e.g. means) or other basic estimates (e.g. regression coefficient) AND variation (e.g. standard deviation) or associated estimates of uncertainty (e.g. confidence intervals)
☐	☒	For null hypothesis testing, the test statistic (e.g. F, t, r) with confidence intervals, effect sizes, degrees of freedom and P value noted<br>*Give P values as exact values whenever suitable.*
☒	☐	For Bayesian analysis, information on the choice of priors and Markov chain Monte Carlo settings
☒	☐	For hierarchical and complex designs, identification of the appropriate level for tests and full reporting of outcomes
☒	☐	Estimates of effect sizes (e.g. Cohen's d, Pearson's r), indicating how they were calculated

Our web collection on statistics for biologists contains articles on many of the points above.

Software and code

Policy information about availability of computer code

Data collection	The microbiome data analyzed in this work are all from published studies. No software was used for data collection.
Data analysis	For taxonomic profiling of shotgun metagenomic sequencing data with reference genomes, we used MetaPhlAn2 (version 2.7; https://github.com/biobakery/MetaPhlAn). For de novo taxonomic profiling of shotgun metagenomic sequencing data without reference genomes, we used MOCAT pipeline (version 2.0; https://mocat.embl.de/), which includes FastX (version 0.013) for quality trimming and filtering of reads, SOAPdenovo (version 1.05) for assembling high quality reads into scaftigs , MetaGeneMark (version 2.8) for predicting genes on scaftigs, BLAT (in MOCAT version 2.0) for clustering the predicted genes, soap.coverage (version 2.7.7) for calculation of gene-length normalized base counts, and MGS-Canopy-Algorithm (https://github.com/fplaza/mgs-canopy-algorithm) for co-abundance clustering. All the other data analysis was performed in MATLAB R2016b.

For manuscripts utilizing custom algorithms or software that are central to the research but not yet described in published literature, software must be made available to editors and reviewers. We strongly encourage code deposition in a community repository (e.g. GitHub). See the Nature Research guidelines for submitting code & software for further information.

Data

Policy information about availability of data

All manuscripts must include a data availability statement. This statement should provide the following information, where applicable:

- Accession codes, unique identifiers, or web links for publicly available datasets
- A list of figures that have associated raw data
- A description of any restrictions on data availability

The microbiome metagenomic data analyzed in this work are all from published studies: HMP (Sequence data are available from the HMP DACC, http://hmpdacc.org); MetaHIT (Sequence data are available from the European Nucleotide Archive under the accession code ERP002061, https://www.ebi.ac.uk/

metagenomics/studies/ERP002061); FMT study of Li et al. (Sequence data are available from the European Nucleotide Archive under the accession code PRJEB12357, https://www.ebi.ac.uk/ena/browser/view/PRJEB12357); FMT study of Smillie et al. (Sequence data are available from the European Nucleotide Archive under the accession code PRJEB23524, https://www.ebi.ac.uk/ena/browser/view/PRJEB23524). The HMP reference genomes are available from the Integrated Microbial Genomes and Microbiome (IMG/M) database (https://img.jgi.doe.gov/). The KEGG Orthologs and pathways are available at KEGG database (https://www.genome.jp/kegg/). The Clusters of Orthologous Groups of proteins are available at https://www.ncbi.nlm.nih.gov/research/cog-project/. The MicroBial Genome Database is available at http://mbgd.genome.ad.jp/. Example data analyzed in this work are available at https://github.com/liangtian85/FR. Source data are provided with this paper.

Field-specific reporting

Please select the one below that is the best fit for your research. If you are not sure, read the appropriate sections before making your selection.

- [x] Life sciences
- [ ] Behavioural & social sciences
- [ ] Ecological, evolutionary & environmental sciences

For a reference copy of the document with all sections, see nature.com/documents/nr-reporting-summary-flat.pdf

Life sciences study design

All studies must disclose on these points even when the disclosure is negative.

Sample size	The microbiome data analyzed in this work are all from published studies. The original experiments and corresponding power analysis have been reported in previous publications. (1) Human Microbiome Project (HMP)[1,41,42]. We analyzed the shotgun metagenomic sequencing data of the human microbiome samples from HMP. We focused on 6 body sites in 5 areas: the gut (one site: stool (549 samples)); the nasal cavity (one site: anterior nares (87 samples)); the oral cavity (2 sites: buccal mucosa (368 samples) and tongue dorsum (418 samples)); the skin (1 site: retroauricular crease (36 samples)); the vagina (1 sites: posterior fornix (52 samples)). (2) Metagenomics of the Human Intestinal Tract (MetaHIT)[4,43]. We analyzed the shotgun metagenomic sequencing data of fecal samples from 177 healthy adults from MetaHIT. (3) Fecal microbiota transplantation (FMT) study of Li et al.[8] A cohort of 5 subjects (metabolic syndrome patients) received a single allogenic FMT from one of three lean donors unrelated to the recipients. Stool samples were collected from the donors (3 samples) and 5 recipients before FMT (5 samples) and after FMT at the 2nd, 14th, 42nd, and 84th days (20 samples). (4) FMT study of Smillie et al.[9] The cohort consist of 19 recurrent C. difficile patients. Feces from one of four donors were transplanted to each patient. Stool samples were collected from the donors (6 samples) and the recipients before FMT (19 samples), and in follow-up visits ranging from 1 day to 4 months after FMT (40 samples).
Data exclusions	For FR calculation with reference genomes, samples with less than 5 strains with known genomes were excluded. For FR calculation without reference genomes (de novo method), no data were excluded.
Replication	Results were tested against different taxonomic profiling pipelines, different genome annotation methods, different microbial genome databases, different definitions of genome functional distance, and different measures of diversity.
Randomization	Four GCN randomization schemes and three microbial composition randomization schemes were adopted to evaluate the impact of GCN structure and microbial composition on within-sample FR.
Blinding	Blinding was not applicable to this work (not a clinical trial).

Reporting for specific materials, systems and methods

We require information from authors about some types of materials, experimental systems and methods used in many studies. Here, indicate whether each material, system or method listed is relevant to your study. If you are not sure if a list item applies to your research, read the appropriate section before selecting a response.

Materials & experimental systems

n/a	Involved in the study	
[x]	[ ]	Antibodies
[x]	[ ]	Eukaryotic cell lines
[x]	[ ]	Palaeontology and archaeology
[x]	[ ]	Animals and other organisms
[x]	[ ]	Human research participants
[x]	[ ]	Clinical data
[x]	[ ]	Dual use research of concern

Methods

n/a	Involved in the study	
[x]	[ ]	ChIP-seq
[x]	[ ]	Flow cytometry
[x]	[ ]	MRI-based neuroimaging
